# Acetylation-dependent USP7-TRIM25 axis drives oncogenic progression in non-small cell lung cancer

**DOI:** 10.1038/s41419-025-08034-9

**Published:** 2025-10-06

**Authors:** Jian Yang, Zhike Chen, Wenxuan Hu, Weibiao Zeng, Zhe Lei, Xin Tong, Qifan Li, Gaomeng Luo, Kang Hu, Zhimeng Chen, Zeyi Liu, Chang Li, Chun Xu, Cheng Ding, Hong-Tao Zhang, Jun Zhao

**Affiliations:** 1 Department of Thoracic Surgery, The First Affiliated Hospital of Soochow University, Suzhou Medical College of Soochow University, Suzhou, Jiangsu Province China; 2 Institute of Minimally Invasive Thoracic Cancer Therapy and Translational Research, Soochow University, Suzhou, Jiangsu Province China; 3 Department of Thoracic Surgery, Shanghai General Hospital, Shanghai Jiao Tong University School of Medicine, Hongkou District, Shanghai, China; 4 Department of Pathology, The First Affiliated Hospital of Soochow University, Suzhou, Jiangsu Province China; 5 Department of Pulmonary and Critical Care Medicine, The First Affiliated Hospital of Soochow University, Suzhou, Jiangsu Province China; 6 Department of Medical Genetics, School of Basic Medical Sciences, Suzhou Medical College of Soochow University, Suzhou, Jiangsu Province China; 7 Suzhou Key Laboratory for Molecular Cancer Genetics, Suzhou, Jiangsu Province China

**Keywords:** Oncogenes, Acetylation, Ubiquitylation, Non-small-cell lung cancer

## Abstract

Tripartite motif containing 25 (TRIM25), an E3 ubiquitin ligase that plays an important role in bioprocesses, is frequently elevated in malignant tumors. However, it remains unclear how TRIM25 protein expression is regulated in non-small cell lung cancer (NSCLC). Here, we find that TRIM25 is hyper-expressed in NSCLC tissues and associated with poor prognosis of NSCLC patients. Both in vitro and in vivo experiments indicate that TRIM25 facilitates tumor proliferation and metastasis. Mechanistically, acetylation is identified as a critical post-translational modification (PTM) regulating TRIM25 protein stability in NSCLC. The lysine acetyltransferase cAMP-responsive element-binding (CREB)-binding protein (CBP) mediates acetylation of TRIM25 at lysine 392, which is counteracted by the deacetylase Sirtuin 7 (SIRT7). Notably, the acetylation of TRIM25 enhances its interaction with ubiquitin specific peptidase 7 (USP7), resulting in reduced ubiquitination of TRIM25. In summary, our study reveals a novel acetylation modification site, thus providing new insights into an epigenetic regulation of TRIM25 in human cancer, and suggesting that pharmacological inhibition of TRIM25 acetylation is a potential anti-tumor strategy.

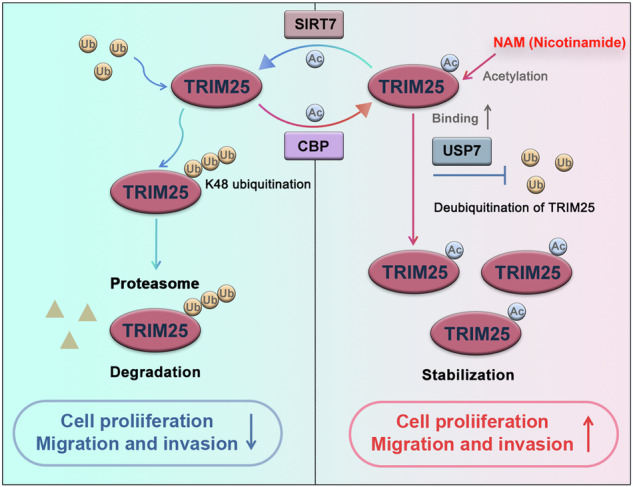

## Introduction

Lung cancer is the leading cause of cancer-related deaths worldwide, non-small cell lung cancer (NSCLC) accounting for approximately 85% of all lung cancer cases [1, 2]. NSCLC is well-known for its rapid progression and increased aggressiveness, particularly in advanced stages. Currently, new medical treatment including targeted therapy and immunotherapy have made great strides [3, 4]. However, the prognosis for NSCLC patients remains poor [5]. Therefore, it is of critical clinical importance to investigate the underlying mechanisms of NSCLC growth and metastasis, as well as to identify potential biomarkers and therapeutic targets.

Lysine acetylation modification is a dynamic and reversible post-translational modification (PTM) of proteins, mediated by lysine acetyltransferase and deacetylases [6]. Initially, many researches focused on histone acetylation within the nucleus, where it plays a crucial role in regulating gene transcription and expression. However, advancements in mass spectrometry have revealed that lysine acetylation occurs extensively on non-histone proteins in the nucleus, cytoplasm, and other organelles. Furthermore, it has been found that an abnormal non-histone protein acetylation state is closely associated with the onset and progression of a variety of diseases, including tumors [7]. Studies have shown that acetylation modifications have a significant impact on the localization [8, 9], activity [10, 11], interaction [12, 13], and stability of non-histone proteins [14, 15]. Particularly, acetylation can crosstalk with other PTMs, such as competing for the same lysine modification site, or affecting phosphorylation and ubiquitination at other sites [16, 17]. In brief, these robust functions highlight the important clinical significance of acetylation modifications, which are expected to provide new promising directions for cancer research and treatment.

TRIM25 is a multifunctional member of the tripartite motif containing protein family, distinguished by its involvement in a variety of cellular processes [18]. Recent research indicates that high expression of TRIM25 significantly contributes to cancer cell proliferation [19], metastasis [20], and drug resistance [21]. For example, TRIM25 targets the Keap1-Nrf2 pathway through its E3 ubiquitin ligase activity, thereby promoting the progression of laryngeal squamous cell and hepatocellular carcinomas [22, 23]. In prostate cancer, TRIM25 modulates p53 signaling by interacting with G3BP2, which enhances tumor cell growth [24]. TRIM25 also regulates the PRMT1/c-MYC pathway by targeting the splicing factor NONO, and promotes the growth and invasion of glioblastoma cells [25]. Additionally, TRIM25 inhibits the binding of the E3 ubiquitin ligase TRAF6 to EZH2, thereby stabilizing EZH2 and promoting oxaliplatin resistance in colorectal cancer [26]. We previously reported that TRIM25 activates the AKT-mTOR signaling pathway by polyubiquitinating PTEN in NSCLC [27, 28]. Although numerous studies have demonstrated that TRIM25 functions as a human cancer-associated protein in multiple dimensions, there is still less understanding of the mechanisms by which TRIM25 itself is regulated. In particular, whether acetylation regulates TRIM25 protein expression warrants further investigation.

In this study, we demonstrate that acetylation attenuates TRIM25 ubiquitination, consequently enhancing its protein stability in NSCLC cells. We identify lysine 392 as the predominant acetylation site of TRIM25, which is dynamically modulated by the acetyltransferase CBP and the deacetylase SIRT7. Functional analyses reveal that acetylation critically governs the oncogenic properties of TRIM25. Importantly, acetylated TRIM25 displays a significantly higher binding affinity for USP7 than its deacetylated form. These findings delineate a novel regulatory crosstalk between acetylation and ubiquitination in controlling TRIM25 protein abundance, thereby unveiling a potential therapeutic strategy for NSCLC driven by acetylated TRIM25.

## Results

### TRIM25 protein level is upregulated in NSCLC and is associated with poor prognosis

**Fig. 1:**
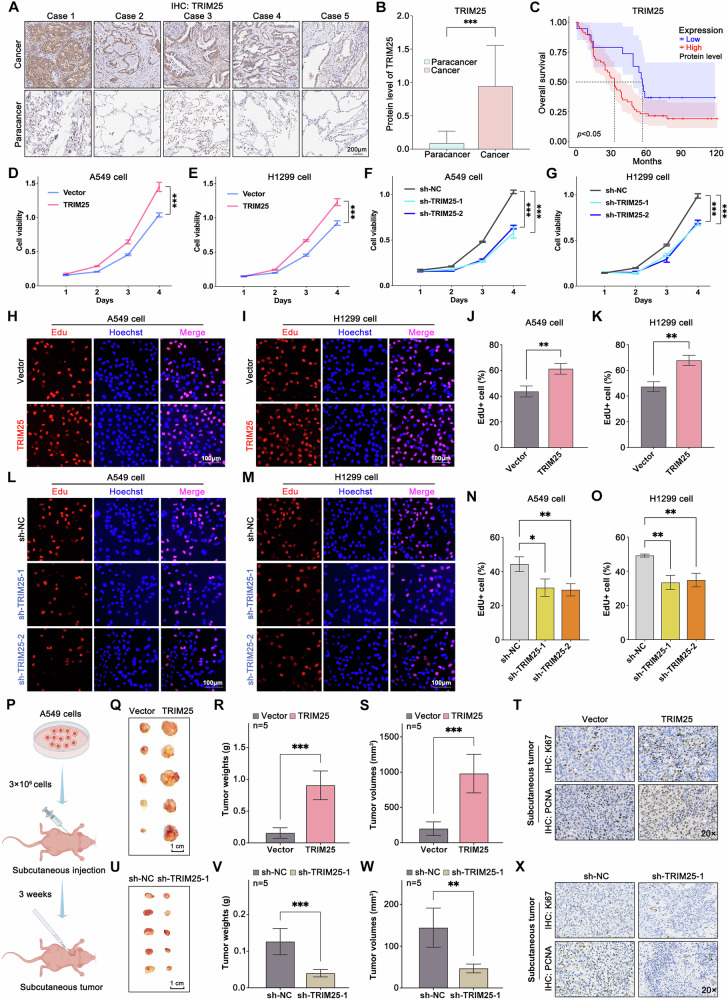
TRIM25 is highly expressed in NSCLC and affects cell growth in vitro and in vivo. **A** NSCLC microarray characterized TRIM25 expression in cancer and paracancer tissues. **B** Comparison of expression scores for cancer (n = 79) and paracancerous (n = 82) tissues. **C** High expression of TRIM25 was associated with poor prognosis in NSCLC patients. **D**–**G** Overexpression of TRIM25 increased NSCLC cell viability, while knockdown of TRIM25 inhibited NSCLC cell viability. **H**–**O** Edu assays indicated that overexpression of TRIM25 promoted the proliferation of NSCLC cells, whereas knockdown of TRIM25 decreased the proliferation ability. **P** Flowchart of the subcutaneous tumor experiments. **Q**–**S** Vector-A549 and TRIM25-A549 cells were injected subcutaneously into nude mice, respectively, and the tumors were removed at the end of the experiment to take photographs and record the weight and volume. **T** IHC results of the Vector group and the TRIM25 group. **U**–**W** sh-NC-A549 and sh-TRIM25-1-A549 cells were injected subcutaneously into nude mice, respectively, and the tumors were removed at the end of the experiment to take photographs and record the weight and volume. **X** IHC results of the sh-NC and the sh-TRIM25-1 group.

To elucidate the expression levels of TRIM25 protein in NSCLC patients, IHC staining was performed on the formalin-fixed paraffin-embedded (FFPE) tissue microarray. As shown in Fig. 1A, B, TRIM25 protein levels were significantly elevated in NSCLC tissues compared to paracancerous tissues. This trend is further confirmed by the CPTAC database (Fig. S1A) on the UALCAN website [29].

Next, we aimed to evaluate the clinical significance of TRIM25 expression in patients with NSCLC. As displayed in Fig. 1C, patients with high TRIM25 expression had worse overall survival (OS) than those with low TRIM25 expression. In summary, these data demonstrate that TRIM25 is significantly upregulated in NSCLC tissues and could serve as a potential prognostic marker for patients with NSCLC.

### TRIM25 facilitates NSCLC cell growth in vitro and in vivo

We then investigated the pathological roles of TRIM25 in NSCLC tumorigenesis. First, we generated NSCLC cell lines with stably overexpressing and silencing of TRIM25 (Fig. S1B–E). The results of CCK8 assays showed that overexpression of TRIM25 increased NSCLC cell viability, whereas knockdown of TRIM25 with shRNAs decreased cell viability (Fig. 1D–G). In addition, the percentage of EdU-positive nuclei increased markedly upon TRIM25 overexpression, demonstrating a marked enhancement in the proliferation of NSCLC cells (Fig. 1H–K). Conversely, the knockdown of TRIM25 significantly inhibited the proliferation of NSCLC cells. (Fig. 1L–O). These in vitro findings demonstrate that TRIM25 facilitates cell proliferation.

To further verify the observed tumor-promoting functions of TRIM25 in vivo, we subcutaneously injected NSCLC cells overexpressing TRIM25 or empty vector as a negative control into nude mice (n = 5 mice per group) for growth analysis. After 3 weeks, the tumors were excised and measured (Fig. 1P, Q). Interestingly, the results of orthotopic xenograft model showed that TRIM25 overexpression promoted the growth of NSCLC cells (Fig. 1R, S). The expression of Ki67 and PCNA in the tumors was upregulated in the TRIM25 group as detected by IHC analysis (Fig. 1T). In a parallel experiment, we injected sh-NC or sh-TRIM25-1 cells into nude mice and removed the tumors after 3 weeks (Fig. 1U). Tumors in the sh-TRIM25-1 group exhibited significantly reduced weight and volume compared to those in the sh-NC group (Fig. 1V, W). IHC analysis confirmed that tumors in the sh-TRIM25-1 group exhibited reduced expression of Ki67 and PCNA (Fig. 1X). These findings collectively demonstrate that TRIM25 significantly enhances the growth of NSCLC cells.

### TRIM25 promotes epithelial mesenchymal transformation (EMT), migration and invasion, and in vivo metastasis of NSCLC cells

**Fig. 2:**
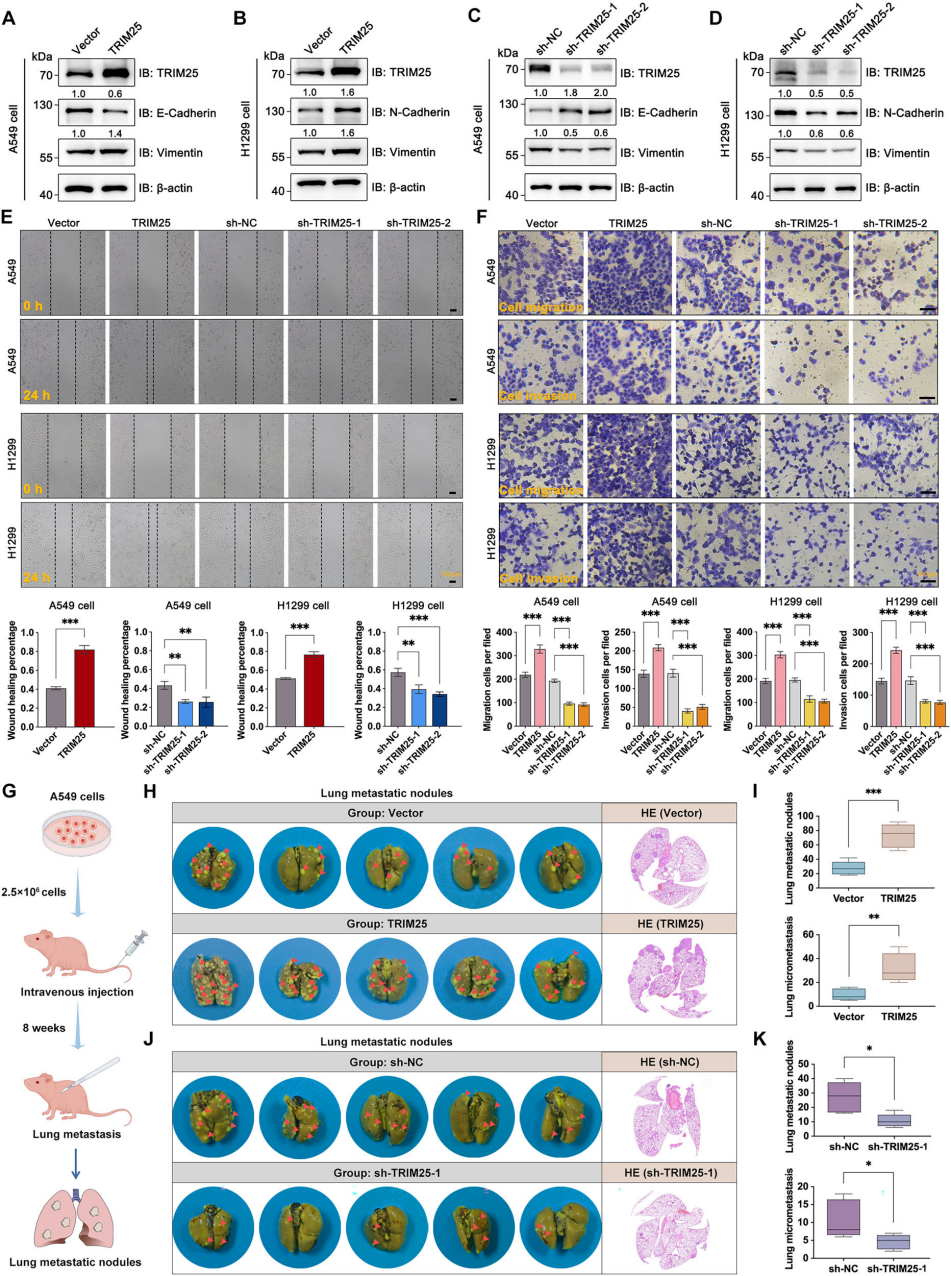
TRIM25 facilitates EMT, migration and invasion, and in vivo metastasis of NSCLC cells*.* **A**, **B** Overexpression of TRIM25 promoted EMT in NSCLC. **C**, **D** Knockdown of TRIM25 inhibited EMT in NSCLC. **E** Scratch assays confirmed that overexpression of TRIM25 promoted migration of NSCLC cells, while knockdown of TRIM25 inhibited migration of NSCLC cells. **F** The transwell assays indicated that overexpression of TRIM25 promoted the migration and invasion of NSCLC cells, whereas knockdown of TRIM25 inhibited the migration and invasion of NSCLC cells. **G** Flowchart of the lung metastasis models. **H**, **I** Vector-A549 and TRIM25-A549 cells were injected into the tail vein of nude mice, respectively, and the lungs were removed at the end of the experiment to take photographs and record the lung metastatic nodules. After HE staining, we further counted the lung micro-metastases. **J**, **K** sh-NC-A549 and sh-TRIM25-1-A549 cells were injected into the tail vein of nude mice, respectively, and the lungs were removed at the end of the experiment to take photographs and record the lung metastatic nodules. After HE staining, we further counted the lung micro-metastases.

Cancer progression is frequently associated with metastasis, in which EMT possesses a crucial and initial role [30]. Therefore, we assessed the expression levels of EMT-related markers in NSCLC cells to investigate the impact of TRIM25 on EMT. As depicted in Fig. 2A–D, TRIM25 overexpression in NSCLC cells increased the levels of the mesenchymal markers, including N-cadherin and Vimentin, while decreasing the level of the epithelial marker E-cadherin, as compared to the Vector cells. Conversely, TRIM25 knockdown resulted in reduced N-cadherin and Vimentin levels, whereas E-cadherin expression was elevated relative to the sh-NC cells. Furthermore, we found that overexpression of TRIM25 in NSCLC cells exhibited enhanced migration and invasion, while TRIM25 knockdown significantly impeded these behaviors (Fig. 2E, F).

To assess the effect of TRIM25 on NSCLC cell metastasis in vivo, we administered Vector or TRIM25 overexpressing cells into the tail veins of nude mice (Fig. 2G, H). After 8 weeks, the mice were euthanized, and their lungs were removed, fixed in Bouin’s solution, and subsequently photographed to visualize metastatic nodules. Following that, HE staining was performed to identify and quantify micro-metastases. Notably, the TRIM25 group exhibited a significantly larger amount of pulmonary metastatic nodules and micro-metastases than the Vector group (Fig. 2I). Further, sh-NC and sh-TRIM25-1 cells were injected into the tail veins of nude mice according to the same protocols. The results indicated that TRIM25 silencing led to a significant reduction in the number of lung metastases compared to the sh-NC group (Fig. 2J, K). Altogether, our findings suggest that TRIM25 is required for NSCLC growth and metastasis.

### TRIM25 is acetylated and deacetylated by CBP and SIRT7, respectively

To determine the mechanisms underlying TRIM25 upregulation in NSCLC, we first evaluated the genomic alterations, including methylation status, copy number variation (CNV), and single nucleotide polymorphism (SNP) of TRIM25 based on the data derived from GSCA platform [31]. In the TCGA dataset, no correlation was observed between TRIM25 methylation and mRNA expression (Fig. S2A). Furthermore, TRIM25 CNV was not significantly associated with altered TRIM25 expression in NSCLC tissues and TRIM25 mutation was rarely detected (Fig. S2B, C). Therefore, we used the PhosphoSitePlus [32] website to predict post-translational modifications that may lead to TRIM25 overexpression in NSCLC. Interestingly, we found that little is known about how acetylation regulated TRIM25 apart from ubiquitination and phosphorylation.

**Fig. 3:**
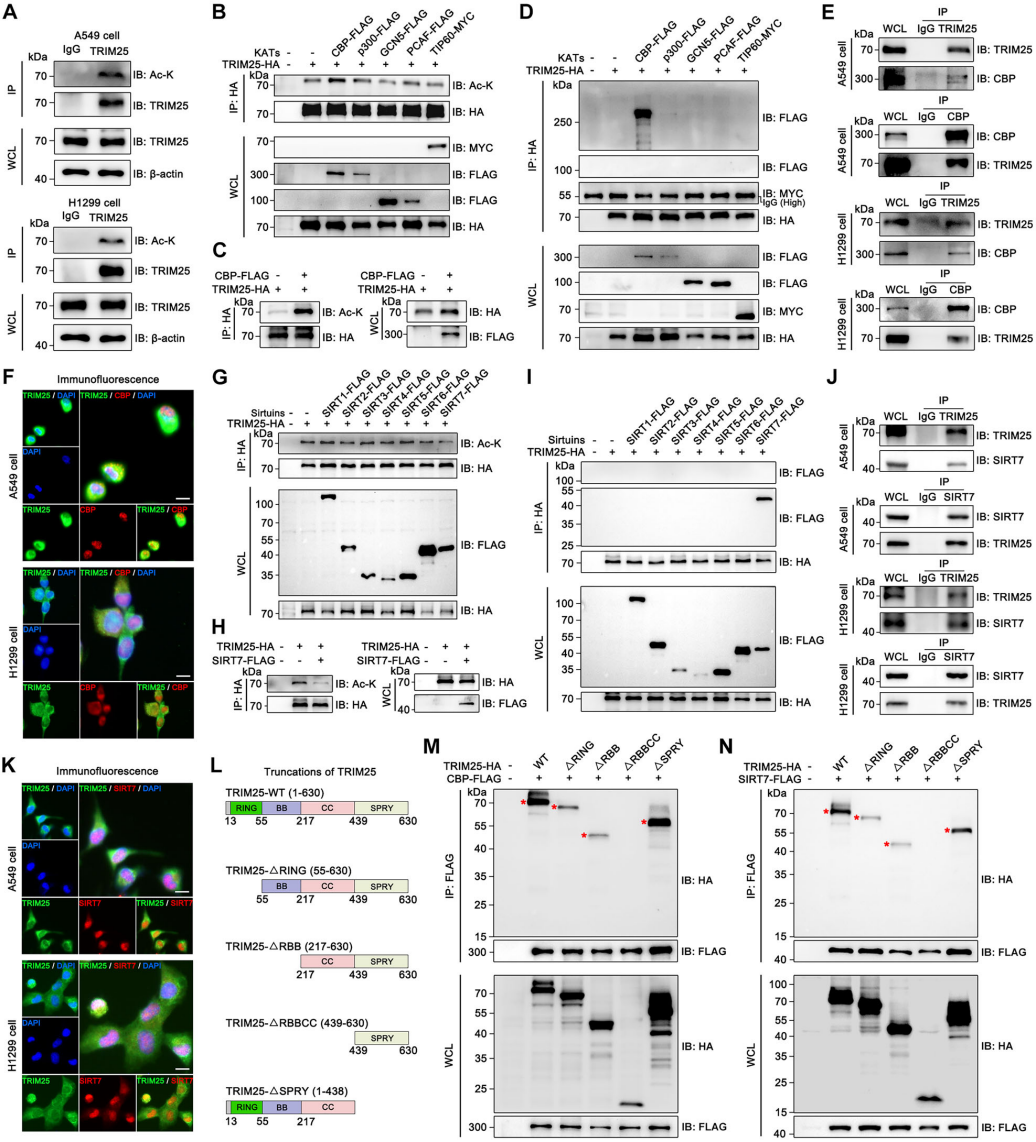
TRIM25 is acetylated and deacetylated by CBP and SIRT7, respectively. **A** Endogenous IP was performed in A549 and H1299, and TRIM25 acetylation was detected. **B** Five acetyltransferases were co-transfected with the TRIM25 as indicated in 293 T cells, and acetylation of TRIM25 was detected (KATs, lysine acetyltransferases). **C** CBP and TRIM25 were transfected in 293 T cells as indicated to detect alterations in TRIM25 acetylation. **D** 293 T cells were transfected as indicated to detect protein interactions. **E** Endogenous interaction between TRIM25 and CBP was detected in A549 and H1299 cells. **F** The co-localization of TRIM25 and CBP in NSCLC cells was detected by IF. **G** Plasmids were co-transfected in 293 T cells as indicated to detect acetylation of TRIM25. **H** SIRT7 and TRIM25 were transfected in 293 T cells as indicated to detect alterations in TRIM25 acetylation. **I** Seven plasmids of the Sirtuin family (SIRT1-7) were co-transfected with the TRIM25 plasmid, respectively, and co-IP was performed to detect protein interactions. **J** Endogenous interaction between TRIM25 and SIRT7 was detected in NSCLC cells. **K** The co-localization of TRIM25 and SIRT7 in NSCLC cells was detected by IF. **L** Four truncations of TRIM25 were designed. **M** Four truncations of TRIM25 were co-transfected with CBP into 293 T cells, respectively, to detect interacting structural domain. **N** Four truncations of TRIM25 were co-transfected with SIRT7 into 293 T cells, respectively, to detect interacting structural domain.

To confirm this point, we performed endogenous IP using an anti-TRIM25 antibody and found that TRIM25 can be acetylated in both A549 and H1299 cells (Fig. 3A). We further identified the lysine acetyltransferase required for TRIM25 acetylation. As shown in Fig. 3B, C, CBP significantly increased the acetylation of TRIM25 compared to p300, GCN5, PCAF, and TIP60. To confirm whether CBP is an interaction partner of TRIM25, we performed co-IP experiments (Fig. 3D). The results showed that CBP bound to TRIM25 at both endogenous (Fig. 3E) and exogenous levels (Fig. S3A, B). Additionally, IF experiments confirmed the co-localization of TRIM25 with CBP in NSCLC cells (Fig. 3F).

Given that TRIM25 is regulated by acetyltransferases, we sought to determine which deacetylase specifically modulates TRIM25. Studies have shown that deacetylases include the Zn^2+^-dependent HDAC family and the NAD^+^-dependent Sirtuins (SIRT) family [33, 34]. Therefore, we transfected cells as outlined in Fig. S4A and stimulated them with Trichostatin A (TSA, inhibitor of the HDAC family) and Nicotinamide (NAM, inhibitor of the SIRT family). We found that NAM but not TSA upregulated TRIM25 acetylation. Notably, in NSCLC cells, we also noted that NAM increases TRIM25 protein expression (Fig. S4B). This implied that acetylation may be involved in TRIM25 protein regulation. NAM is known to inhibit the SIRT family of deacetylases, suggesting that a member of this family might mediate TRIM25 deacetylation. Excitingly, we identified SIRT7 as a negative regulator that downregulates TRIM25 acetylation (Fig. 3G, H). Furthermore, our experiments demonstrated that SIRT7 interacts with TRIM25 (Fig. 3I). This interaction was further confirmed by exogenous IP (Fig. S5A, B), endogenous IP (Fig. 3J), and IF experiments (Fig. 3K).

Then, to elucidate the specific structural regions of TRIM25 that interact with CBP and SIRT7, we designed four TRIM25 truncation constructs for this study (Fig. 3L). They are TRIM25-ΔRING, TRIM25-ΔRBB, TRIM25-ΔRBBCC, and TRIM25-ΔSPRY. Our results indicated that the interaction between TRIM25 and the two above-mentioned proteins was disrupted when the CC region of TRIM25 is deleted (Fig. 3M, N). This finding indicated that the CC region was crucial for TRIM25 acetylation. In conclusion, these data clearly support the idea that CBP and SIRT7 are molecular switches responsible for the acetylation and deacetylation of TRIM25.

### TRIM25 is acetylated primarily at the K392 residue

**Fig. 4:**
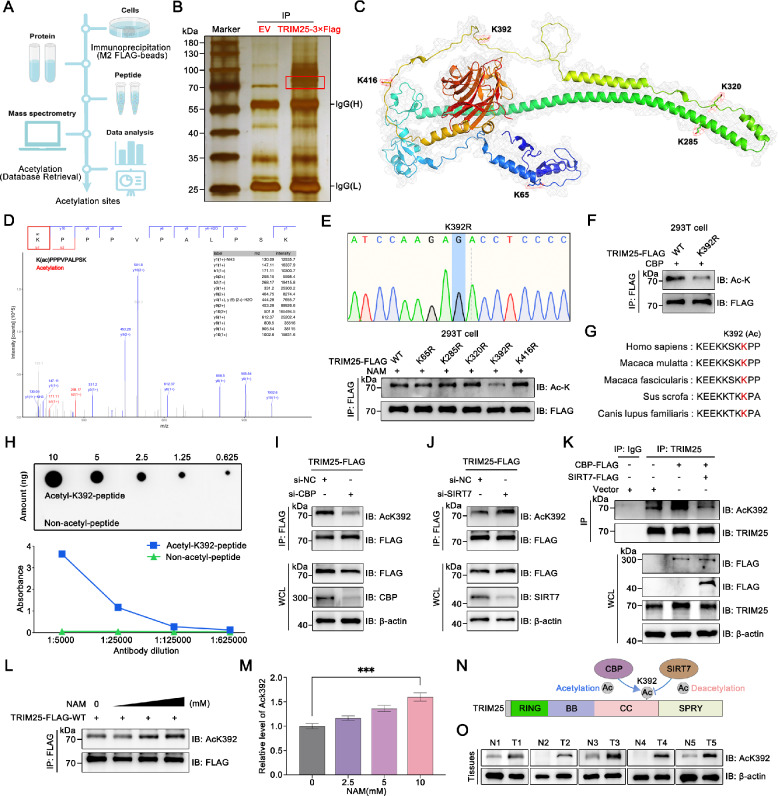
CBP and SIRT7 act as a molecular switch to mediate K392 acetylation and deacetylation of TRIM25. **A** Identification of the TRIM25 acetylation sites by mass spectrometry. **B** Silver staining confirmed the enrichment of TRIM25 in the IP sample. **C** Five potential acetylation sites were identified. **D** Mass spectrum of K392(ac). **E** Five TRIM25 mutant plasmids were constructed based on TRIM25-WT. After sequencing, the above plasmids were transfected into 293 T cells and stimulated with NAM to detect TRIM25 acetylation. **F** CBP was co-transfected with TRIM25-WT or TRIM25-K392R plasmids to detect TRIM25 acetylation. **G** Conservativeness analysis of K392 site. **H** Dot-blot and ELISA assays to validate AcK392-TRIM25 antibody. **I** Detection of TRIM25 acetylation after knockdown of CBP. **J** Detection of TRIM25 acetylation after knockdown of SIRT7. **K** Exogenous overexpression of CBP or SIRT7 in A549 cells, and detected acetylation of TRIM25 with AcK392-TRIM25 antibody. **L** Acetylation at the K392 site was regulated by NAM in a dose-dependent manner (Concentration of NAM: 0 mM, 2.5 mM, 5 mM, 10 mM). **M** Relative protein level of Ack392. **N** CBP and SIRT7 dynamically regulated K392 site acetylation. **O** Proteins were extracted from cancer tissues and paired paracancerous normal tissues of five NSCLC patients, and protein levels of AcK392-TRIM25 were detected by WB.

To identify the primary lysine site involved in TRIM25 acetylation, we purified overexpressed TRIM25-FLAG and performed liquid chromatography-tandem mass spectrometry analysis (Fig. 4A, B). As shown in Fig. 4C, the TRIM25 structure was simulated by AlphaFold [35] and visualized by PyMol software. After peptide identification and database retrieval, we pinpointed five potential lysine sites, and displayed these spectrums (Fig. 4D and Fig. S6A). Subsequently, we constructed five TRIM25 mutant plasmids (lysine mutated to arginine) to mimic the deacetylated state of TRIM25. The mutant plasmids used above were sequenced and the results were all correct. To determine which site is predominantly regulated by acetyltransferase, we transfected 293 T cells with mutant plasmids and stimulated them with NAM for 6 h. We found no significant up-regulation of acetylation levels in the TRIM25-K392R mutant, suggesting that the K392 may be the major acetylation site of TRIM25 (Fig. 4E and Fig. S6B). Further, this trend was verified in cells overexpressing CBP (Fig. 4F).

K392 site is conserved across species (Fig. 4G). Therefore, we generated a specific antibody for AcK392-TRIM25, which was validated using dot-blot and ELISA assays (Fig. 4H). Based on this customized antibody, we further confirmed that CBP catalyzed acetylation of the K392 site of TRIM25, and this trend could be counteracted by SIRT7 in A549 cells (Fig. 4I–K). In addition, we found that the acetylation level of the K392 site was regulated by NAM in a dose-dependent manner (Fig. 4L, M). Collectively, these results confirm that CBP and SIRT7 dynamically regulate TRIM25 acetylation at the K392 site (Fig. 4N).

To comparatively evaluate TRIM25 acetylation status in malignant and normal cells, we performed WB analysis using two normal bronchial epithelial cell lines and two NSCLC cell lines. Our results revealed a marked upregulation of TRIM25 acetylation at the K392 site in NSCLC cells compared to normal counterparts (Fig. S7A, B). Furthermore, this observation was corroborated in clinical tissue specimens (Fig. 4O). These data collectively indicate that TRIM25 acetylation is significantly elevated in tumor cells, implicating its potential role in NSCLC progression.

### Acetylation stabilizes TRIM25 by inhibiting its K48 ubiquitination modification

**Fig. 5:**
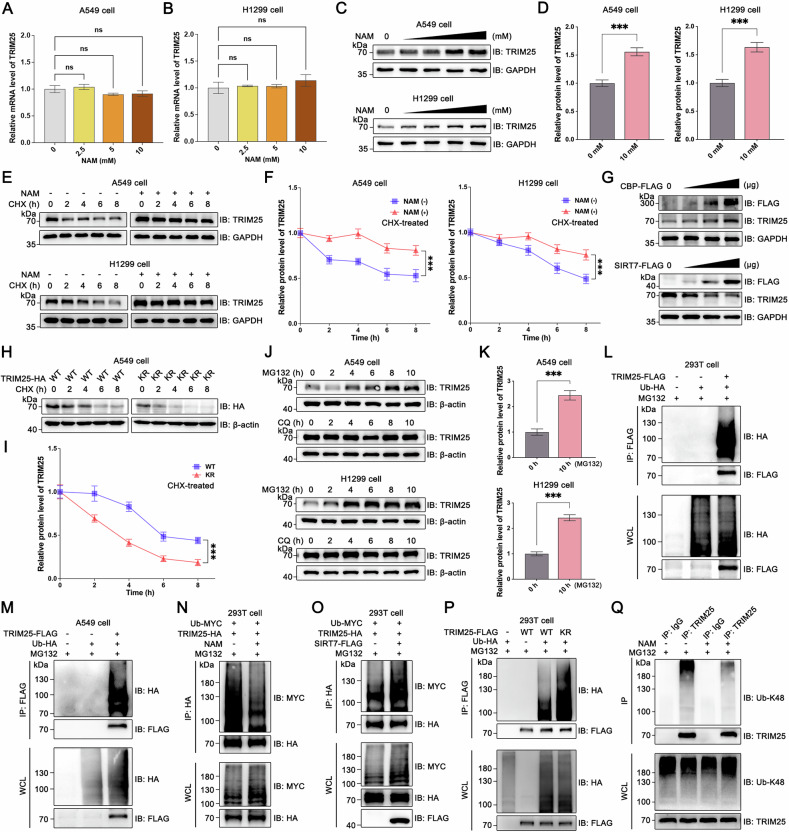
Acetylation of TRIM25 affects its protein expression level by inhibiting K48 ubiquitination. **A**, **B** RT-qPCR was performed to detect the mRNA expression level of TRIM25 after NAM stimulation. **C**, **D** Protein expression of TRIM25 was assayed after NAM stimulation in A549 and H1299 cells (Concentration of NAM: 0 mM, 2.5 mM, 5 mM, 10 mM, 20 mM). **E**, **F** The groups without or with NAM were set up in NSCLC cells, and CHX (100 μM) were added to detect TRIM25 protein expression, respectively. **G** Protein expression of TRIM25 was assayed after gradient overexpression (0 μg, 0.5 μg, 1 μg, 2 μg) of CBP or SIRT7 in NSCLC cells. **H**, **I** TRIM25-WT and TRIM25-KR cells were treated with CHX to detect the trend of TRIM25 protein expression, respectively (KR is an abbreviation for K392R in this paper). **J**, **K** NSCLC cells were treated with a time gradient of MG132 (10 μM) or CQ (50 μM), respectively, to detect TRIM25 expression. **L**, **M** TRIM25-FLAG and Ub-HA plasmids were transfected in 293 T and A549 cells as indicated to test whether TRIM25 could be ubiquitinated. **N** Ubiquitination of TRIM25 was detected after NAM stimulation in 293 T cells. **O** Ubiquitination of TRIM25 was detected after transfection of SIRT7 in 293 T cells. **P** The TRIM25-WT and TRIM25-KR plasmids were co-transfected with Ub-HA, respectively, to detect the effect of TRIM25-KR on TRIM25 ubiquitination. **Q** Endogenous IP for detecting the effect of NAM stimulation on TRIM25-K48 ubiquitination.

Considering that TRIM25 can be acetylated, it is important to determine the impact of this modification on TRIM25. As we all know, acetylation modifications play an important role in regulating protein stability [15], so based on the observed results we hypothesized whether K392 acetylation could affect TRIM25 stability. To verify this, we treated A549 and H1299 cells with NAM for 6 h. Surprisingly, RT-qPCR showed no corresponding rise in TRIM25 mRNA levels (Fig. 5A, B), while TRIM25 protein levels increased following NAM stimulation (Fig. 5C, D). This suggests that acetylation can regulate TRIM25 at the protein level.

Further, we conducted cycloheximide (CHX) experiments to assess TRIM25 stability in NSCLC cells after NAM treatment (Fig. 5E). These experiments revealed that NAM stimulation enhanced TRIM25 protein stability compared to the control group (Fig. 5F). Additionally, stepwise overexpression of CBP and SIRT7 demonstrated that CBP stabilized TRIM25, while SIRT7 destabilized TRIM25 (Fig. 5G). To determine whether the K392 site is involved in this stabilization mechanism, we compared the half-lives of TRIM25-WT and TRIM25-K392R (Fig. 5H). We found that TRIM25-WT exhibited greater stability compared to TRIM25-K392R (Fig. 5I). This finding suggests that TRIM25 is less stable and more prone to degradation when deacetylated at the K392 site.

Protein degradation primarily occurs through the proteasome system or the lysosomal pathway [36]. To determine which pathway is predominantly responsible for TRIM25 degradation in NSCLC, we treated NSCLC cells with MG132, chloroquine (CQ) and bafilomycin A1 (Baf-A1), respectively (Fig. 5J, K and Fig. S8A). Our results demonstrated that TRIM25 protein levels increased following MG132 treatment, whereas no significant change was observed upon CQ or Baf-A1 treatment, indicating that the proteasome pathway is a major contributor to TRIM25 degradation in NSCLC.

Since the ubiquitin-proteasome is the most common mechanism of protein degradation, so to confirm whether TRIM25 undergoes ubiquitination, we conducted IP and WB experiments in both 293 T and A549 cells (Fig. 5L, M). As shown by the WB experiments, NAM treatment significantly reduced TRIM25 ubiquitination (Fig. 5N), while SIRT7 overexpression led to increased ubiquitination levels (Fig. 5O). Given the observed effects of acetylation on TRIM25 stability, we consider that acetylation affects TRIM25 ubiquitination.

To further investigate the role of acetylation at the K392 site in regulating TRIM25 ubiquitination, we transfected Ub-HA, TRIM25-WT and TRIM25-K392R plasmids in A549 cells. The results revealed that ubiquitination of TRIM25 increased after deacetylation at K392 site (Fig. 5P). Subsequently, to verify whether acetylation affects K48 ubiquitination, we performed experiments using an antibody specific for K48 ubiquitination. Notably, our results demonstrated that K48 ubiquitination modification of TRIM25 was significantly reduced under NAM stimulation (Fig. 5Q). In addition, considering the crucial regulatory function of K63 ubiquitination in protein activity, we conducted additional IP experiment to examine whether acetylation at the K392 site modulates K63 ubiquitination of TRIM25. However, our experimental result showed that K392 acetylation did not influence TRIM25’s K63 ubiquitination (Fig. S8B). In conclusion, our studies demonstrate that acetylation at K392 inhibits proteasomal degradation by reducing K48 ubiquitination, thereby stabilizing TRIM25.

### Deacetylation of TRIM25 suppresses NSCLC tumor progression

**Fig. 6:**
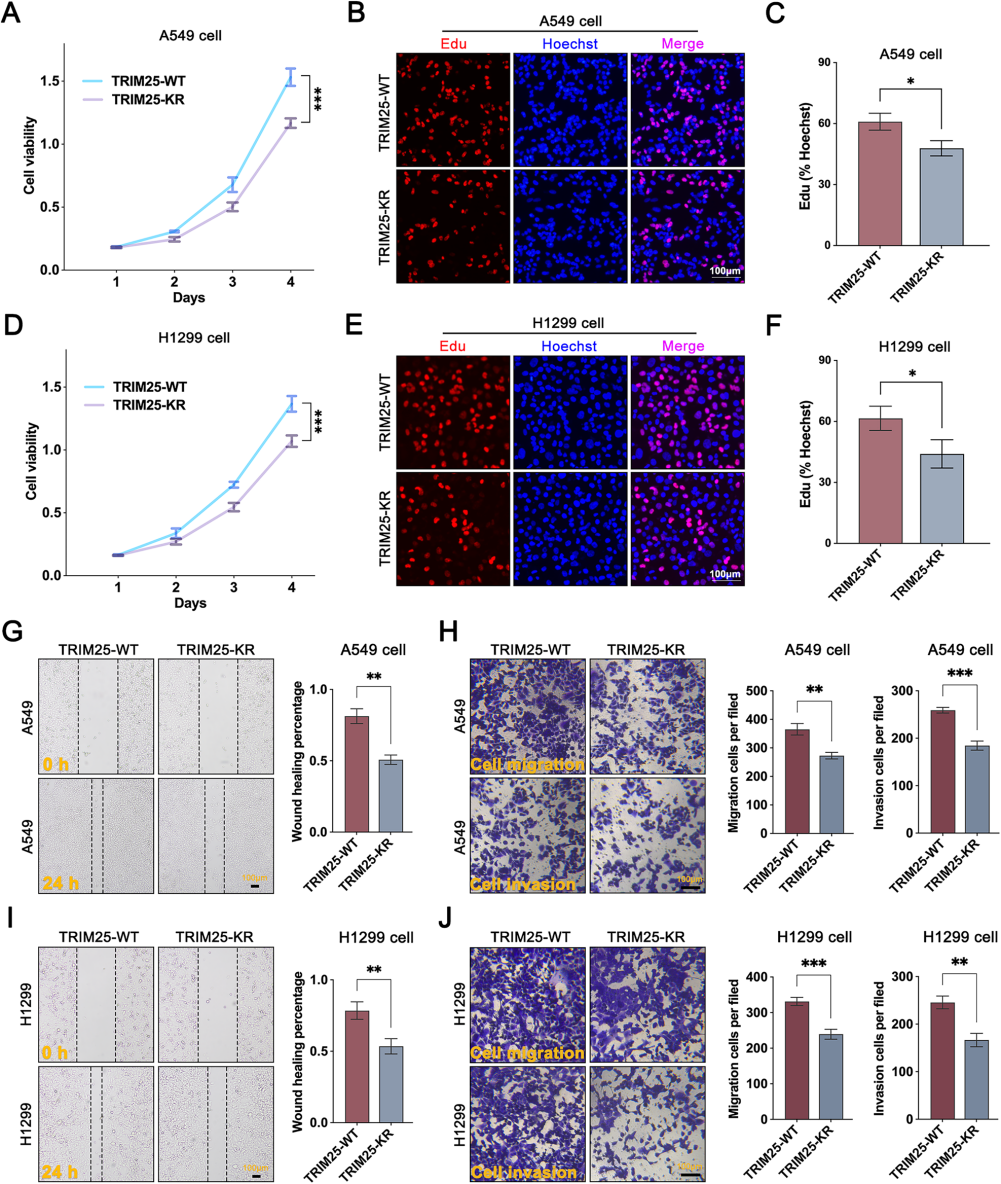
Deacetylation modification of TRIM25 suppresses its oncogenic function. **A**–**C** TRIM25-WT and TRIM25-K392R were exogenously overexpressed in A549 cells, respectively, followed by CCK-8 and EdU assays to detect cell viability and proliferation ability. **D**–**F** TRIM25-WT and TRIM25-K392R were exogenously overexpressed in H1299 cells, respectively, followed by CCK-8 and EdU assays to detect cell viability and proliferation ability. **G**, **H** The effects of TRIM25-WT and TRIM25-K392R on the migration and invasion ability of A549 cells were compared using scratch assays and transwell assays. **I**, **J** The effects of TRIM25-WT and TRIM25-K392R on the migration and invasion ability of H1299 cells were compared using scratch assays and transwell assays.

To elucidate the regulatory mechanism of acetylation modification in the progression of NSCLC, this study systematically investigated the functional roles of TRIM25-WT and its acetylation site mutant (TRIM25-K392R) through comprehensive in vitro and in vivo experiments. In vitro experiments demonstrated that, compared to the TRIM25-WT group, overexpression of TRIM25-K392R significantly suppressed the proliferative capacity of A549 and H1299 cells, as consistently validated by CCK-8 cell viability assays and EdU proliferation assays (Fig. 6A–F). Furthermore, wound-healing and transwell migration/invasion assays revealed that TRIM25-K392R overexpression markedly attenuated the migratory and invasive abilities of NSCLC cells compared with overexpression of TRIM25-WT (Fig. 6G–J). These findings collectively indicate that deacetylation of TRIM25 effectively inhibits its oncogenic activity.

To validate the in vitro results, we established a subcutaneous xenograft tumor model in nude mice using A549 cells stably overexpressing TRIM25-WT or TRIM25-K392R. In vivo data showed that, compared to the wild-type control, TRIM25-K392R (deacetylated form) significantly suppressed tumor progression (Fig. S9A–C), which was highly consistent with the in vitro observations. Based on these findings, we propose that targeted modulation of TRIM25 acetylation, particularly at the K392 site, may represent a novel therapeutic strategy for NSCLC.

### Acetylation of TRIM25 promotes its binding to USP7

**Fig. 7:**
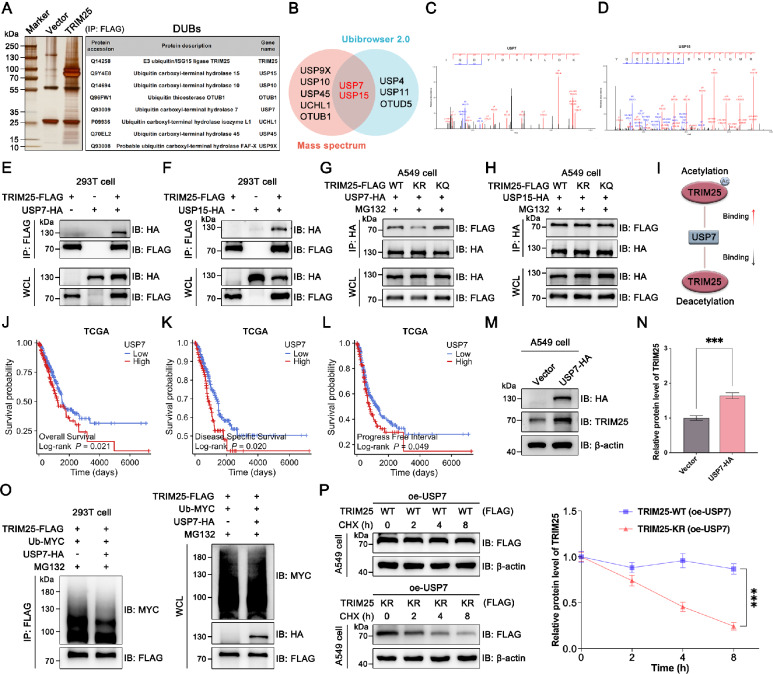
K392 acetylation recruits USP7 to deubiquitinate and stabilize TRIM25. **A** Identification of DUBs by mass spectrometry in A549 cells. **B** Ubibrowser 2.0 database combined with mass spectrometry to identify two potential DUBs. **C**, **D** Mass spectrums of USP7 and USP15. **E** In 293 T cells, TRIM25 was co-transfected with USP7 to detect protein interactions. **F** In 293 T cells, TRIM25 was co-transfected with USP15 to detect protein interactions. **G** TRIM25-WT, TRIM25-KR, and TRIM25-KQ were co-transfected with USP7 in A549 cells to clarify whether acetylation could regulate USP7. **H** TRIM25-WT, TRIM25-KR, and TRIM25-KQ were co-transfected with USP15 to clarify whether acetylation could regulate USP15. **I** Binding of USP7 to TRIM25 was regulated by acetylation. **J**–**L** High expression of USP7 was associated with poor prognosis in TCGA database. **M**, **N** Overexpression of USP7 stabilized protein expression of TRIM25. **O** IP experiments confirmed that USP7 can deubiquitinate TRIM25. **P** TRIM25-WT and TRIM25-KR plasmids were transfected into USP7 stably overexpressing A549 cells respectively, and CHX were added to detect the protein expression of TRIM25.

We next explored how acetylation regulates ubiquitination of TRIM25. Given that TRIM25 expression is tightly controlled by ubiquitination, we performed mass spectrometry and identified multiple potential deubiquitination enzymes (DUBs) in A549 cells (Fig. 7A). In addition, we predicted the DUBs of TRIM25 using Ubibrowser 2.0 database [37]. We hypothesized that acetylation regulated TRIM25 protein expression by recruiting DUBs. As shown in the Venn diagram (Fig. 7B), two DUBs (USP7 and USP15) were finally identified as targets after screening (Fig. 7C, D).

Subsequently, we constructed USP7-HA and USP15-HA plasmids and co-transfected with TRIM25-FLAG plasmid for co-IP experiments, respectively. The results showed that both USP7 and USP15 interacted with TRIM25 in 293 T cells (Fig. 7E, F) as well as A549 cells (Fig. S10A, B). To further determine whether acetylation of TRIM25 could recruit the two DUBs mentioned above, we transfected cells as shown and tested the binding ability between TRIM25 and two DUBs, respectively (Fig. 7G, H). The results showed that there were no major differences in the binding ability of the three TRIM25 constructs to USP15. On the other hand, we found that USP7 had the lowest binding capacity to TRIM25-K392R (deacetylated state), and the highest binding capacity to TRIM25-K392Q (acetylation mimic) (Fig. 7I).

Then, we further used endogenous IP assays to confirm the interaction of TRIM25 with USP7 in A549 and H1299 cells (Fig. S11A). In addition, by downloading and analyzing the TCGA-LUAD database, we found that USP7, as a poor prognostic factor, could indeed affect the survival of NSCLC patients (Fig. 7J–L). To further determine whether USP7 could stabilize TRIM25 and synergistically play a role in promoting NSCLC progression, we constructed the A549 cell line overexpressing USP7. It was confirmed by WB experiments that overexpression of USP7 stabilized the expression of TRIM25 (Fig. 7M, N). In addition, the results of co-IP in 293 T and A549 cells also indicated that USP7 could deubiquitinate TRIM25 (Fig. 7O and Fig. S11B). Based on the above results, we designed half-life experiments in which A549 cells (stably overexpressing USP7) were exogenously transfected with TRIM25-WT or TRIM25-K392R. After CHX treatment, we found no significant decrease in protein expression of TRIM25 in TRIM25-WT group cells. However, in TRIM25-K392R group cells, TRIM25 expression was significantly reduced despite the presence of USP7. These results suggested that the stabilizing effect of USP7 on TRIM25 was dependent on the acetylation of TRIM25 (Fig. 7P). In conclusion, these findings suggest that acetylation plays an important role in maintaining high TRIM25 expression in NSCLC, and inhibition of TRIM25 acetylation is expected to down-regulate TRIM25 protein levels and thus inhibit tumor progression.

## Discussion

Protein PTMs are essential mechanisms for the regulation of intracellular protein function, including phosphorylation [38], ubiquitination [39], acetylation [40], methylation [41], hydroxylation [42], glycosylation [43], neddylation [44], dopaminylation [45], lactylation [46, 47], and many other types. They play key roles in numerous biological processes and are associated with tumor progression. Therefore, an in-depth study of PTM, a popular research direction that cannot be ignored, is expected to provide new targets for clinical treatment [48, 49].

TRIM25 has been reported to be associated with antiviral natural immunity and cancer [18, 50, 51]. As an E3 ubiquitin ligase, TRIM25 functions by recognizing and binding to a variety of substrates. Based on our previous findings, TRIM25 is markedly upregulated in NSCLC and inhibits the function of PTEN. Nevertheless, the driving mechanisms of elevated TRIM25 expression remains unclear. To address this gap, we initially explored potential genetic regulatory mechanisms at the DNA level. However, no favorable results were obtained by database analysis. Consequently, we redirected our investigation toward PTMs of TRIM25.

In cancer, existing studies have focused on phosphorylation, neddylation, and ubiquitination of TRIM25. For example, previous research has demonstrated that MAP3K13 phosphorylates the S12 residue of the E3 ubiquitin ligase TRIM25, which decreases its polyubiquitination and subsequent proteasomal degradation [52]. Additionally, neddylation at the K117 site of TRIM25 reduces the spatial obstruction of its RING domain, thereby enhancing TRIM25’s binding to TFEB. This interaction facilitates TFEB’s nuclear translocation and transcriptional activation of autophagy-related genes, ultimately diminishing the sensitivity of triple-negative breast cancer to paclitaxel [53]. In bladder cancer, USP7 stabilizes TRIM25 through deubiquitination, thereby promoting tumor progression [54]. Given the critical involvement of TRIM25 in cancer progression, it is imperative to determine whether additional PTMs, such as acetylation, contribute to its functional regulation. Therefore, this study aims to investigate the acetylation of TRIM25 in NSCLC and to elucidate the underlying molecular mechanisms.

Here, we present the first evidence that TRIM25 undergoes acetylation modification in NSCLC. Building on this discovery, we further investigated whether acetylated TRIM25 plays a role in NSCLC progression. Using IP and WB techniques, we discovered that CBP mediates the K392 acetylation of TRIM25, and this modification is dynamically counteracted by SIRT7. Notably, TRIM25 exhibits elevated expression levels in NSCLC. Considering the pivotal role of PTMs in modulating protein stability, we further investigated whether acetylation influences the stability of TRIM25. We constructed a K392R mutant plasmid and performed half-life experiments, which revealed that deacetylated TRIM25 exhibits significantly reduced protein stability. These results indicate that acetylation at K392 is critical for maintaining the high expression levels of TRIM25.

Lysine acetylation modifications are known to stabilize non-histone proteins through various mechanisms. One way in which acetylation stabilizes proteins is by affecting ubiquitination at the same or other sites. For instance, in colon cancer, TRIB3 acetylation mediated by the acetyltransferase p300 inhibits its ubiquitination [55]. Specifically, p300 acetylates the K240 residue of TRIB3, reducing its binding affinity for the E3 ubiquitin ligase SIAH1 and thereby preventing the ubiquitination and degradation of TRIB3 at the K197 site. Additionally, acetylation of MCL1 at the K40 site recruits the deubiquitinase USP9X, which enhances protein interactions and stabilizes MCL1 by preventing its ubiquitination [17]. This stabilization inhibits apoptosis and promotes colony formation. Furthermore, in lung cancer, acetylation of MOB1 by CBP at K11 site similarly stabilizes the protein [16]. This acetylation modification inhibits MOB1’s interaction with E3 ubiquitin ligases, thus protecting it from proteasomal degradation.

Notably, in this study we demonstrate that TRIM25 is primarily degraded through the ubiquitin-proteasome pathway, and its ubiquitination levels are modulated by acetylation. Specifically, acetylation of TRIM25 inhibits its ubiquitination, whereas deacetylation enhances it. The K392R mutation of TRIM25 exhibits significantly higher ubiquitination levels compared to the WT, indicating that acetylation at the K392 site may modulate TRIM25 stability by influencing ubiquitination at other sites, but not at the same site. In addition, by mass spectrometry and IP experiments, we verify that the deubiquitination modification of TRIM25 by USP7 is regulated by TRIM25-K392 acetylation. These findings provide insight into the elevated expression of TRIM25 in NSCLC, highlighting the importance of acetylation in its regulation.

Despite the contributions of this study, several limitations remain. First, further research is needed to explore the potential for designing targeted drugs against the acetylation site of TRIM25. In addition, given the diverse effects of acetylation modifications on proteins, it remains unclear whether other mechanisms exist, such as regulating TRIM25 to interact with other proteins. In the future, further exploration is required to refine the regulatory mechanism of acetylation modification for TRIM25.

## Materials and methods

### Cell lines and tissue specimens

A549, H1299, HBE, BEAS2B and 293 T cells were purchased from the Cell Bank of the Chinese Academy of Sciences, authenticated by STR profiling and free of mycoplasma contamination. HBE, BEAS2B, A549 and H1299 cells were cultured in RPMI-1640 medium (Gibco) containing 10% fetal bovine serum (FBS; Invitrogen). 293 T cells were cultured in high glucose DMEM medium (Thermo Fisher Scientific) containing 10% FBS. The incubator was set at a temperature of 37 °C with a humidified 5% CO_2_ atmosphere. Five fresh-frozen NSCLC tissues and matched paracancerous tissues were collected after informed consent from patients at the First Affiliated Hospital of Soochow University (Suzhou, China). The inclusion criteria for the samples were patients with a clear pathological diagnosis of NSCLC who had not undergone preoperative treatments such as radiotherapy, chemotherapy, immunotherapy, or targeted therapy.

### Antibodies

The antibodies utilized in this study were: Mouse anti-β-actin (Proteintech, Cat#66009-1-Ig), Mouse anti-TRIM25 (Proteintech, Cat#67314-1-Ig), Rabbit anti-TRIM25 (Proteintech, Cat#12573-1-AP), Rabbit anti-USP7 (Proteintech, Cat#66514-1-Ig), Mouse anti-E-cadherin (BD Biosciences, Cat#610181), Mouse anti-N-cadherin (BD Biosciences, Cat#610920), Mouse anti-Vimentin (Santa Cruz biotechnology, Cat#sc-6260), Mouse anti-GAPDH (ABclonal, Cat#AC002), Rabbit anti-HA-Tag (Cell Signaling Technology, Cat#3724 s), Rabbit anti-FLAG-Tag (Cell Signaling Technology, Cat#14793 s), Mouse anti-MYC-Tag (ABclonal, Cat#AE038), Rabbit anti-MYC-Tag (Cell Signaling Technology, Cat#2278), Rabbit anti-IgG (Cell Signaling Technology, Cat#3900), Mouse anti-IgG (Cell Signaling Technology, Cat#5415), Rabbit anti-Acetylated-Lysine (Cell Signaling Technology, Cat#9441), Rabbit anti-CBP (Cell Signaling Technology, Cat#7389), Rabbit anti-SIRT7 (Proteintech, Cat#12994-1-AP), Rabbit anti-K48-Ubiquitin (ABclonal, Cat#A3606), Rabbit anti-PCNA (ABclonal, Cat#A12427), Rabbit anti-Ki-67 (ABclonal, Cat#A20018), Rabbit anti-AcK392-TRIM25 (Cohesion Biosciences, CKKVSKEEKKSK-K(Ac)-PPP, Cat#K2219).

### Construction of plasmids and transfections

TRIM25 was cloned into pcDNA3.1-HA, pCDH-HA, and pECMV-3×FLAG vectors, respectively. CBP was separately cloned into pcDNA3.1-FLAG and pcDNA3.1 vectors. p300, GCN5, and PCAF were cloned into pcDNA3.1-FLAG vector. TIP60 was cloned into pcDNA3.1-MYC vector. SIRT1, SIRT2, SIRT3, SIRT4, SIRT5, SIRT5, SIRT6 and SIRT7 were cloned into pCDH-FLAG vector. Ub was cloned into pcDNA3.1-MYC and pcDNA3.1-HA vectors, respectively. USP7 was cloned into pcDNA3.1-HA and pCDH-HA vectors, respectively. USP15 was cloned into pcDNA3.1-HA vector. TRIM25 mutation constructs were generated using the Mut ExpressFast Mutagenesis Kit (Vazyme). Truncated CDS domains of TRIM25 were amplified with the corresponding PCR primers (Supplementary Table S1) and cloned into pCDH-HA vector. sh-NC, sh-TRIM25-1, and sh-TRIM25-2 were synthesized by Tsingke and then cloned into the lentiviral vector pLKO.1-puro, respectively. The sequences of shRNAs were listed in Supplementary Table S1. All plasmids were validated with Sanger sequencing. Subsequently, 293 T, A549 or H1299 cells were transiently transfected with the above plasmids using Lipofectamine 3000.

### Real-time quantitative reverse transcriptase PCR (qRT-PCR)

Total RNA was isolated from cells using TRIzol (Thermo Fisher Scientific) according to the manufacturer’s instructions. cDNA was synthesized using Reverse Transcriptase (Vazyme). qRT-PCR was performed on a Roche LightCycler 96 instrument (Roche Diagnostics) using SYBR Green I. Primers for qRT-PCR analysis were listed in Supplementary Table S1. Relative expression of each mRNA was calculated using the ∆∆Ct method. Three biological replicates of each experiment were performed.

### Short interfering RNAs (siRNAs) and RNA interference

The si-CBP and si-SIRT7 were designed and synthesized by Genepharma. A scrambled siRNA (si-NC) served as negative control. The sequences of siRNAs were listed in Supplementary Table S1. Cells were transiently transfected with 100 pmol siRNAs. After 48 h of transfection, cells were collected for further experiments.

### Generation of stable cell lines

Lentiviral pCDH-Vector, pCDH-TRIM25-HA, pCDH-TRIM25-K392R-HA, sh-NC, sh-TRIM25-1, sh-TRIM25-2, and pCDH-USP7-HA were co-transfected with packaging plasmids psPAX2 and pMD2.G for 48 h in 293 T cells. The packaged lentiviruses were collected for infection of NSCLC cells. Stable cells were finally selected with 0.2 μg/ml puromycin (Solarbio).

### Western blot (WB)

WB analyses were performed as we described previously [56], and data were quantified using ImageJ software. Briefly, cells were lysed by RIPA lysis solution (Thermo Fisher Scientific) containing protease cocktail and phosphatase cocktail (Sangon Biotech). After centrifugation, protein samples were mixed with loading buffer and DTT, and boiled at 100 °C for 10 min. Subsequently, protein samples were subjected to SDS-PAGE gel electrophoresis and transferred to nitrocellulose membrane (MERCK). After being blocked for 1 h at room temperature, the membranes were incubated in primary antibody at 4 °C overnight. Subsequently, the membranes were washed three times by TBST and then incubated in secondary antibody for 2 h at room temperature. Finally, the protein was detected by chemiluminescent kit (Fdbio science).

### CCK8 and EdU assays

Stable NSCLC cell lines were plated in 96-well plates. Cell viability assays were performed on 1-, 2-, 3- and 4-days using Cell Counting Kit-8 (CCK8; Beyotime) according to the manufacturer’s instructions. Optical density (OD) values were determined by measuring the absorbance at 450 nm. For the EdU (5-ethynyl-2’-deoxyuridine) assay, cells were seeded into 24-well plates at 5 × 10^4^ cells per well and cultured to the appropriate cell density. The EdU-594 kit (Beyotime) was utilized to assess the proliferation of NSCLC cells. Positive staining for EdU and DAPI was observed under a fluorescence microscope, and EdU ratios were recorded for three random fields.

### Transwell migration and invasion assays

Transwell assays were performed as we described previously [30]. Briefly, 20% FBS medium was placed in each well of the transwell plate (BD Biosciences), and NSCLC cells were resuspended in 1% FBS medium and seeded into migration chambers or Matrigel (Gibco) invasion chambers (8-mm pore, Corning). The transwell plate was placed in 37 °C incubator for 24 h and then removed to fix the migrated or invaded cells. Cells were stained with 0.1% crystal violet and counted for three microscopic fields. Each transwell analysis was repeated three times.

### Wound-healing migration assay

A549 and H1299 cells were inoculated in 6-well plates. After 90% confluency was reached, cells were scratched with the tip of a sterile pipette and continued to be cultured in serum-free medium for 24 h. Three random areas were photographed under the microscope at 0 h and 24 h, respectively. Wound width was analyzed using ImageJ software. Each wound healing analysis was repeated three times.

### Immunohistochemical (IHC) staining

The human NSCLC tissue microarray used in this study were from Shanghai Outdo-Biotech. TRIM25 antibody was used to detect protein expression. The expression of TRIM25 was quantified by evaluating the staining intensity and positive rate after excluding samples that did not meet the pathological diagnostic criteria. A total of 79 cancer tissue samples and 82 adjacent normal tissue samples were finally included. The specific steps were as follows. Tissue sections were soaked 3 times in xylene to completely remove paraffin, and then soaked in anhydrous ethanol for 2 times to remove xylene. The tissue sections were gradually hydrated by soaking them in 90%, 80% and 70% ethanol for 3 min each. Subsequently, the tissue sections were washed with PBS buffer for several minutes to remove residual reagents. Then, tissue sections were placed in antigen repair solution under high temperature and pressure. After cooling naturally, inactivator (3% H_2_O_2_) was added and incubated away from light to remove interfering substances such as endogenous peroxidase. After being permeabilized with 0.25% Triton X-100 for 25 min, the tissue sections were incubated in the blocking solution for 30 min at room temperature. Then, the tissue sections were evenly covered with TRIM25 primary antibody and incubated at 4 °C overnight. The next day, the tissue sections were incubated at room temperature for 30 min using the appropriate secondary antibody, followed by the addition of the color developing solution according to the instructions to observe the results.

### In vivo NSCLC cell growth assays

Four-week-old female BALB/c nude mice were purchased from the Laboratory Animal Center of Soochow University and fed in a specific pathogen-free environment. The Animal Ethics Committee of Soochow University has approved all experimental animal studies.

Mice in each group (randomized grouping, n = 5) were injected with the same type of cells (3 × 10^6^ cells/mouse). Briefly, cells were injected subcutaneously into the right flank of mice. After injection, the nude mice were observed periodically. At the endpoint of the experiment, tumors were harvested to measure the weight and volume. Single-blinding were used for measurement. Tumor weight was taken by balance. Tumor volume was calculated as: (mm^3^) = D × d^2^ × 0.52, where D and d represented the longest and shortest diameters of the tumor, respectively. Subsequently, tumor tissues were fixed with 4% paraformaldehyde and IHC staining (Ki67 and PCNA) was performed on paraffin-embedded sections.

### In vivo metastasis assays

Four-week-old female BALB/c nude mice were purchased from the Laboratory Animal Center of Soochow University and housed under specific pathogen-free conditions. The Animal Ethics Committee of Soochow University has approved all experimental animal studies.

According to the principle of randomization, 5 mice were used in each group. TRIM25-A549 cells, sh-TRIM25-1-A549 cells, and corresponding control A549 cells in 200 μl PBS were injected into the tail vein of mice, respectively (2.5 × 10^6^ cells/mouse). The nude mice were observed periodically, and eight weeks after injection, the mice were euthanized. Single-blinding were used for measurement. Lungs were removed and fixed in Bouin’s solution for metastatic nodule analysis. Subsequently, hematoxylin and eosin (HE) staining were performed to count the number of micro-metastases.

### Immunofluorescence (IF) staining

IF was performed as we described with some modifications [57]. Briefly, Cells on coverslips were fixed in 4% paraformaldehyde for 30 min and permeabilized with 0.5% Triton X-100 for 15 min. The cells were washed with PBS buffer and incubated in 5% BSA for 1 h. Subsequently, the primary antibody was incubated at 4 °C overnight. After washing the cells with PBST buffer, they were incubated with FITC-conjugated anti-mouse secondary antibody or Cy3-conjugated anti-rabbit secondary antibody for 2 h at room temperature. Cell nuclei were counterstained with DAPI. Finally, pictures were taken using the Zeiss fluorescence microscope.

### IP and mass spectrum

Cells in dishes were lysed in ice-cold IP lysate for 30 min. After centrifugation, a portion of the sample was retained as input control (WCL, whole cell lysate). The rest of cell lysates were incubated with magnetic beads carrying antibodies anti-Flag (Sigma) or anti-HA (Thermo Fisher Scientific), or with appropriate antibody-coupled protein A/G magnetic beads. After being washed by the IP buffer, the prepared immunoprecipitate and input can be subjected to WB analysis. In addition, the product after IP can also be used for mass spectrometry (supported by PTM BIO) to obtain specific protein peptides, as well as to identify acetylation sites.

### Protein silver-staining assay

The experiment was performed as previously described [30]. The eluted co-immunoprecipitation (co-IP) products were separated on SDS-PAGE gels and then characterized using a silver staining kit (CWBIO).

### The cancer genome atlas (TCGA) database

TCGA is a public database containing abundant cancer genomic data for 33 major cancer types. For this study, we downloaded the lung adenocarcinoma (LUAD) transcriptome expression data and converted the data to TPM format for normalization. In addition, we downloaded the corresponding clinical information data of the patients from the database. Based on the expression data and prognostic data of cancer patients, we performed the Kaplan–Meier prognostic analysis.

### Statistical analysis

Differences between the two groups were compared by paired or unpaired Student’s *t* test, while one-way ANOVA was used for analyzing data with three or more groups. Data were expressed as means±Standard deviation (SD). The sample size was determined based on previous studies demonstrating consistent effects with similar experimental designs. Protein levels were quantified using ImageJ software and data were analyzed using GraphPad Prism 9 software. *p < 0.05; **p < 0.01; ***p < 0.001.

## Supplementary information

Supplementary Figures S1 to S11

Supplementary Table S1

Source data for Figures

## Data Availability

Source data and reagents are available from the corresponding author upon reasonable request.
