# Supplementary material for: Acetylation-dependent USP7-TRIM25 axis drives oncogenic progression in non-small cell lung cancer
Source: Cell Death Dis. 2025 Oct 6;16(1):695. doi: 10.1038/s41419-025-08034-9 (PMC12501092; doi:10.1038/s41419-025-08034-9)
Supplement: Supplementary file 1 — Supplementary Figures S1 to S11 [file 41419_2025_8034_MOESM1_ESM.pdf]

Supplementary Fig. S1

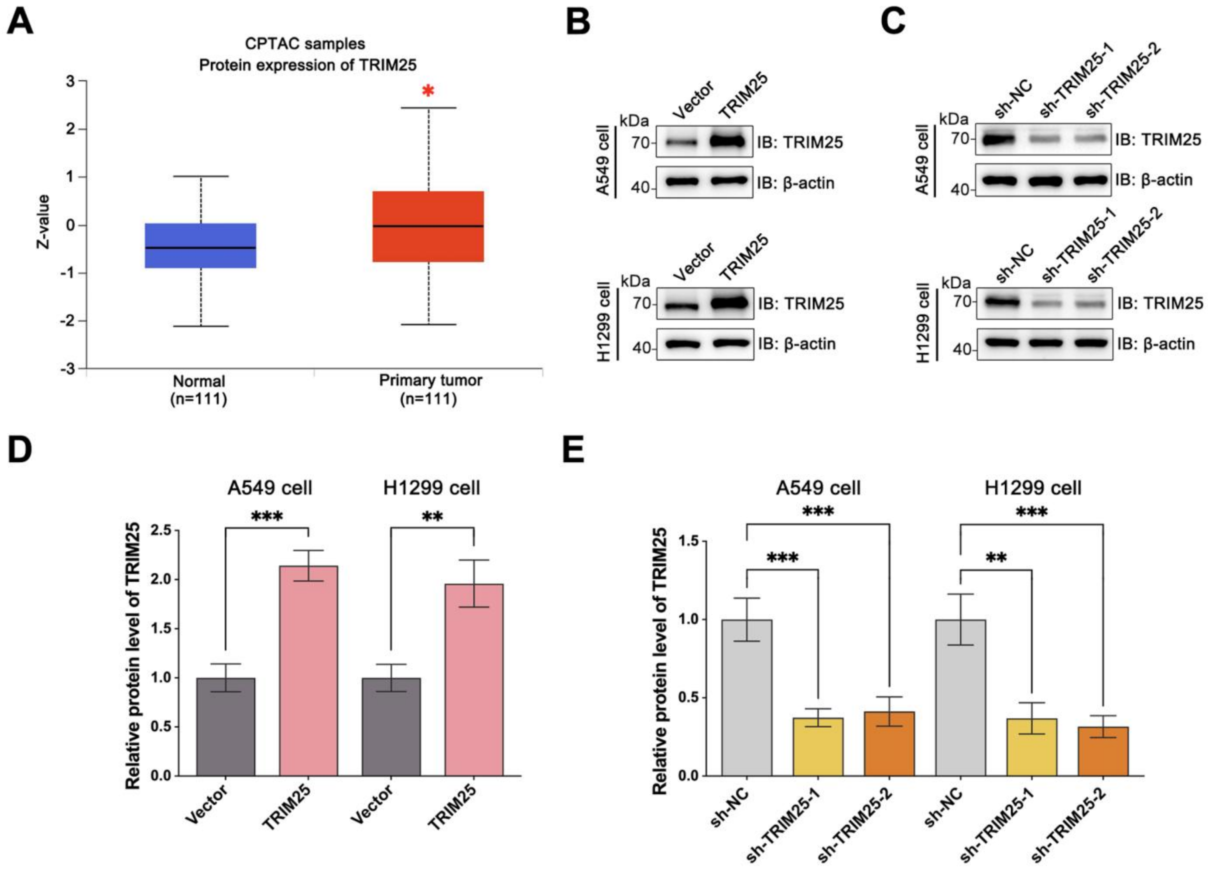

Fig. S1 Analysis of TRIM25 protein levels in NSCLC samples and construction of stable NSCLC cell lines. (A) Protein expression analysis of TRIM25 in the CPTAC-LUAD database. (B) Vector and TRIM25-HA lentivirus were used to infect A549 and H1299 cells, and the overexpression efficacy was detected by western blot. (C) sh-NC, sh-TRIM25-1 and sh-TRIM25-2 lentiviruses were used to infect A549 and H1299 cells, and the knockdown effect was detected by western blot. (D-E) Quantitative protein analysis of cell lines stably overexpressing or knocking down TRIM25.

Supplementary Fig. S2

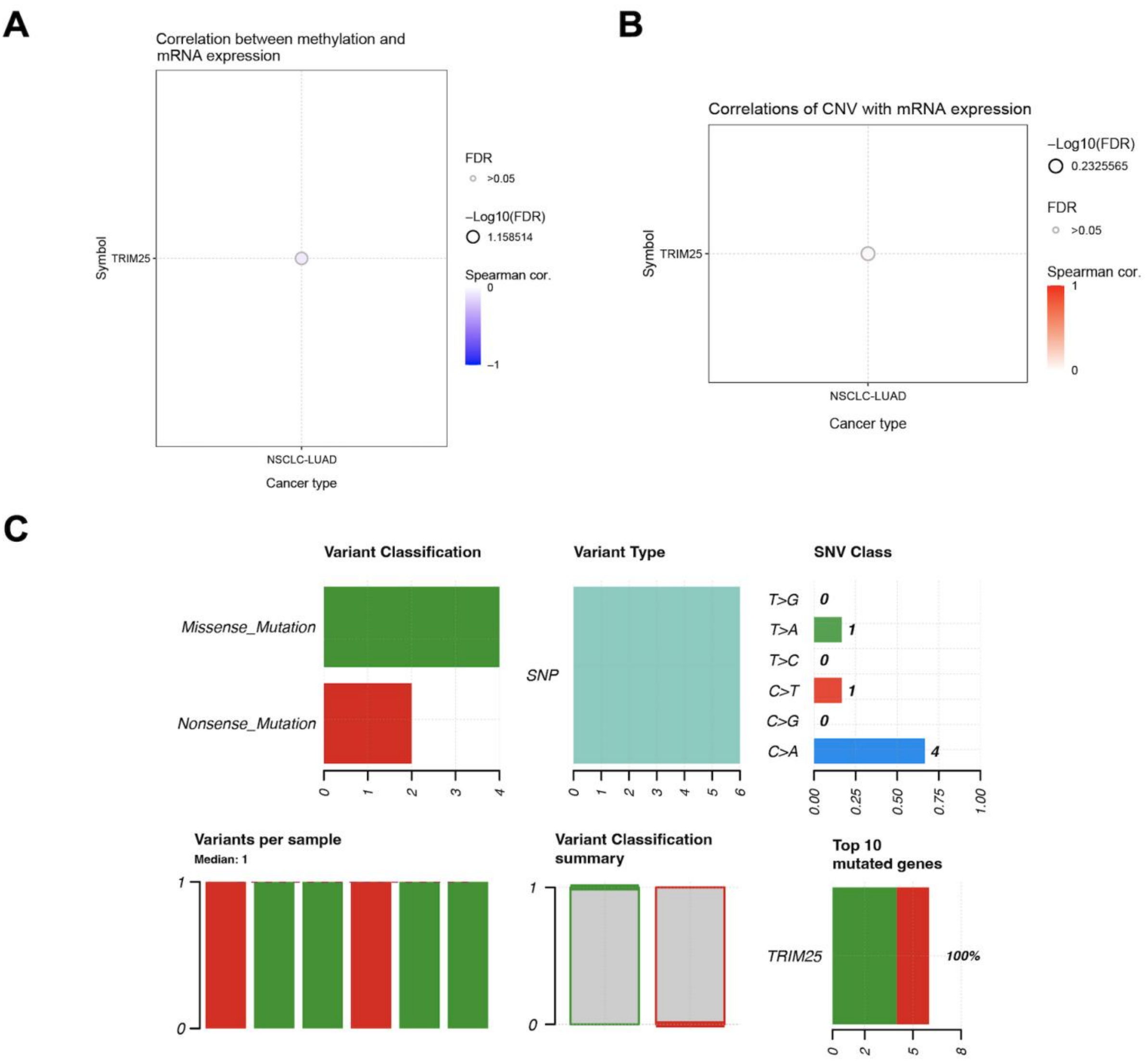

Fig. S2 Methylation status, CNV and SNP analysis of TRIM25. (A) Correlation between methylation and TRIM25 mRNA expression. (B) Correlation between CNV and TRIM25 mRNA expression. (C) Genetic mutation and SNP analysis of TRIM25.

Supplementary Fig. S3

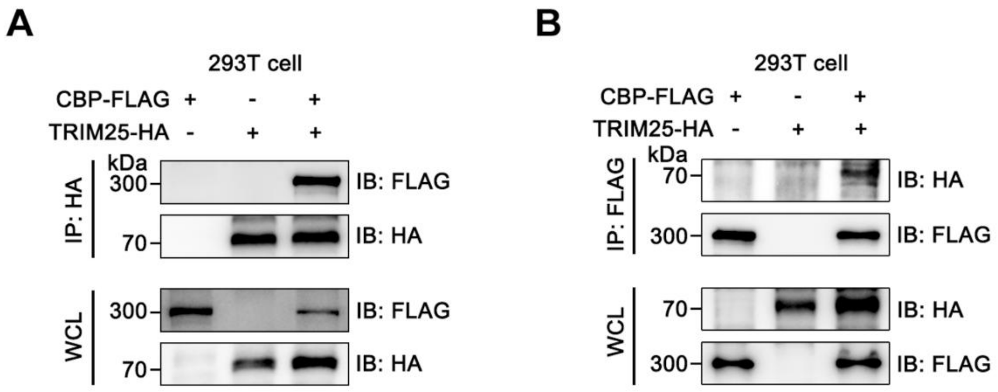

Fig. S3 The interaction of TRIM25 with CBP is identified using exogenous transfection. (A) TRIM25-HA and CBP-FLAG plasmids were transfected into 293T cells, and co-IP assays were performed with anti-HA magnetic beads to detect exogenous interactions. (B) TRIM25-HA and CBP-FLAG plasmids were transfected into 293T cells, and co-IP assays were performed with anti-FLAG magnetic beads to detect exogenous interactions.

Supplementary Fig. S4

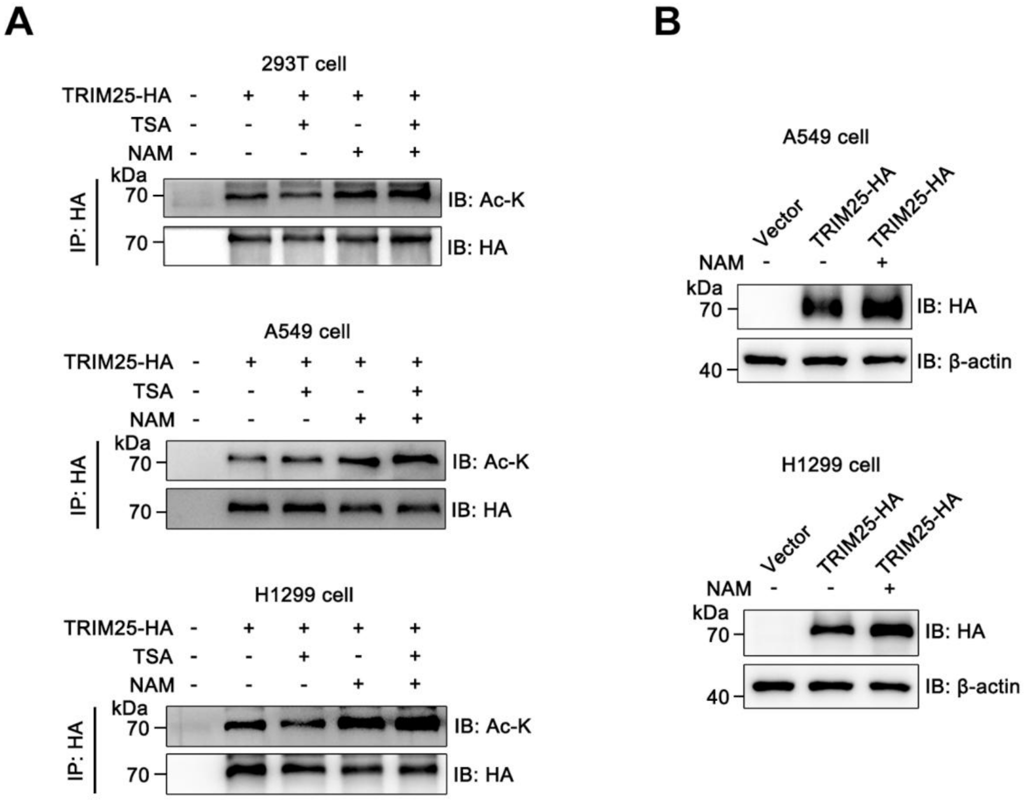

Fig. S4 Acetylation of TRIM25 is regulated by NAM but not TSA. (A) Cells were transfected with TRIM25-HA plasmid and treated with TSA or NAM before protein extraction, followed by IP experiments. (B) NAM was added to A549 and H1299 cells stably overexpressing TRIM25-HA, and the expression of TRIM25-HA was detected by western blot assay.

Supplementary Fig. S5

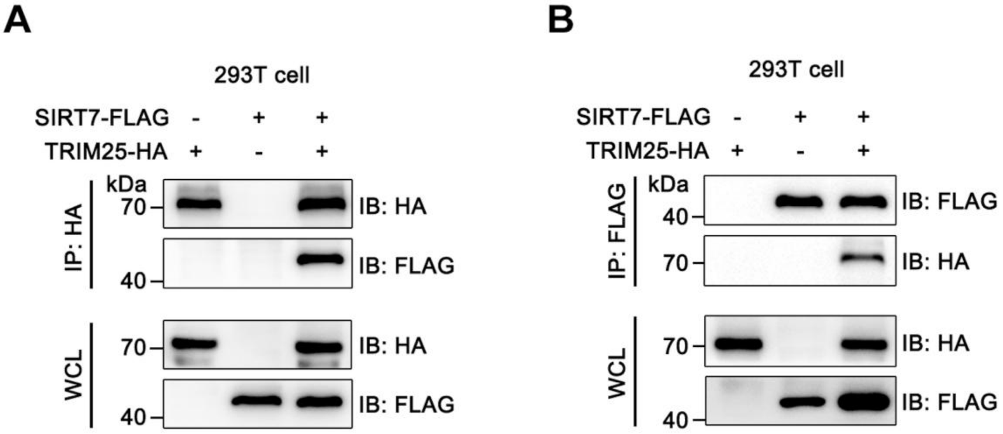

Fig. S5 The interaction of TRIM25 with SIRT7 is identified using exogenous transfection. (A) TRIM25-HA and SIRT7-FLAG plasmids were transfected into 293T cells, and co-IP assays were performed with anti-HA magnetic beads to detect exogenous interactions. (B) TRIM25-HA and SIRT7-FLAG plasmids were transfected into 293T cells, and co-IP assays were performed with anti-FLAG magnetic beads to detect exogenous interactions.

Supplementary Fig. S6

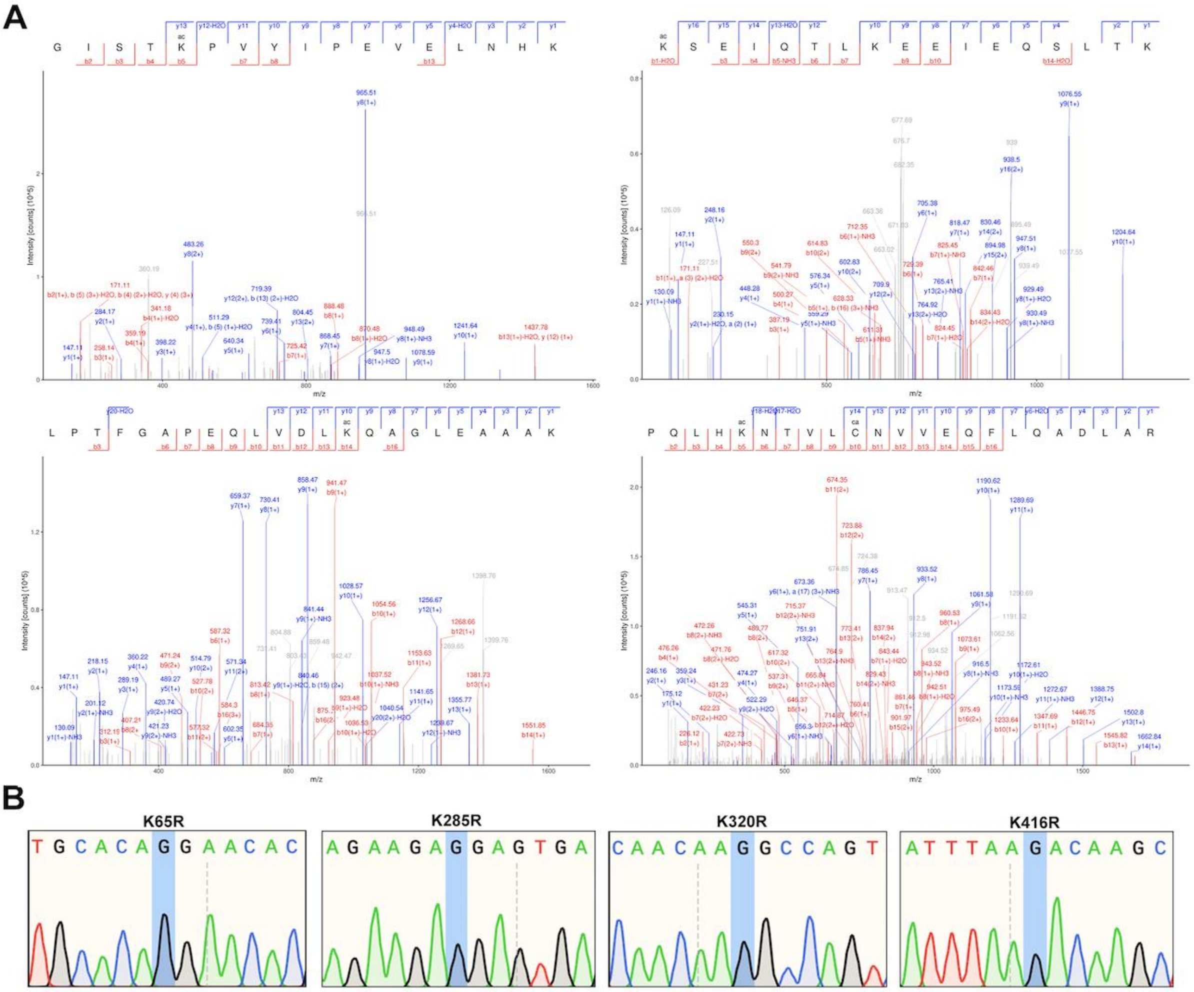

Fig. S6 Construction of TRIM25 mutant vectors. (A) Mass spectrums of the lysine sites (K65, K285, K320, K416). (B) Sequencing results of the mutation sites (K65R, K285R, K320R, K416R).

Supplementary Fig. S7

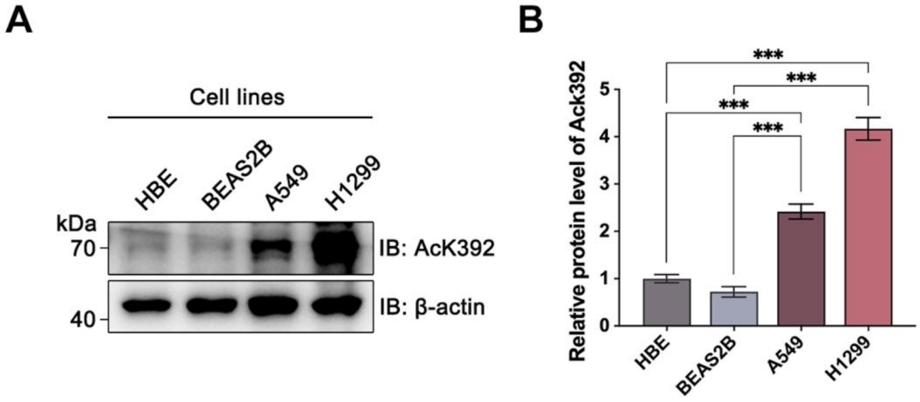

Fig. S7 Protein levels of AcK392-TRIM25 in cell lines. (A-B) Proteins from two normal bronchial epithelial cell lines (HBE and BEAS2B) and two NSCLC cell lines (A549 and H1299) were extracted, and protein levels of AcK392-TRIM25 were detected by western blot.

Supplementary Fig. S8

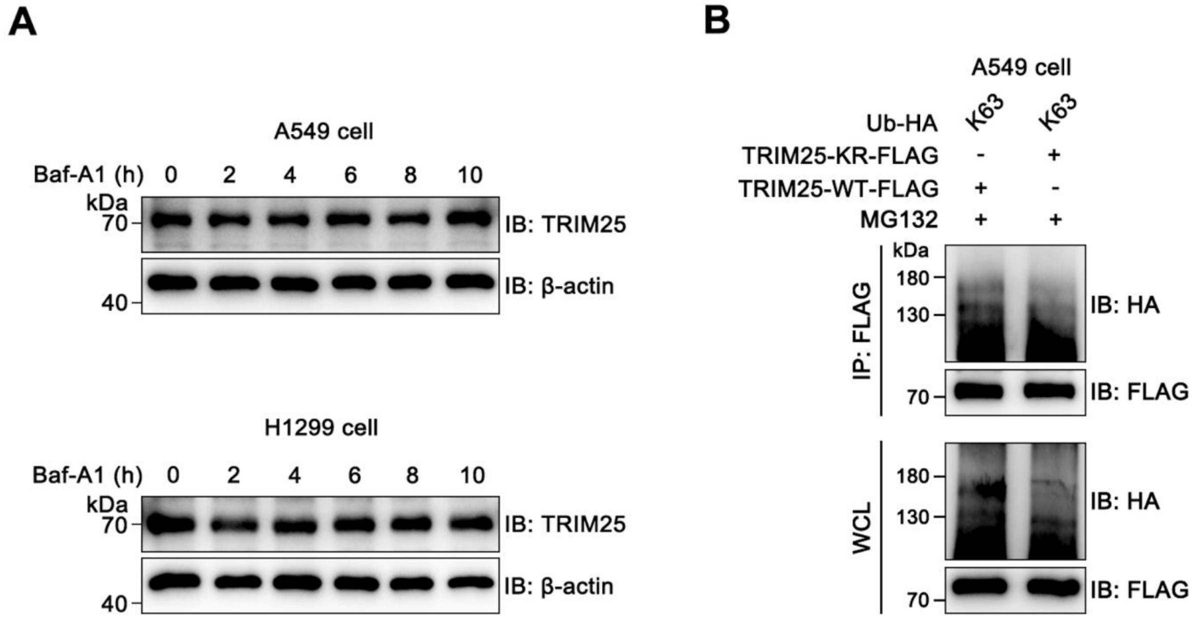

Fig. S8 TRIM25 protein degradation is not regulated by the autophagy-lysosome pathway, and K392 acetylation does not modulate the K63 ubiquitination modification of TRIM25. (A) A549 and H129 cells were treated with the autophagy inhibitor Baf-A1 (10nm), and a time gradient (0, 2, 4, 6, 8, 10h) was set. At the end of the treatment, cellular proteins were extracted and subjected to western blot experiments. (B) Plasmids were transfected in A549 cells, and after 48 hours, IP experiments were performed using anti-FLAG magnetic beads, followed by western blot experiments.

Supplementary Fig. S9

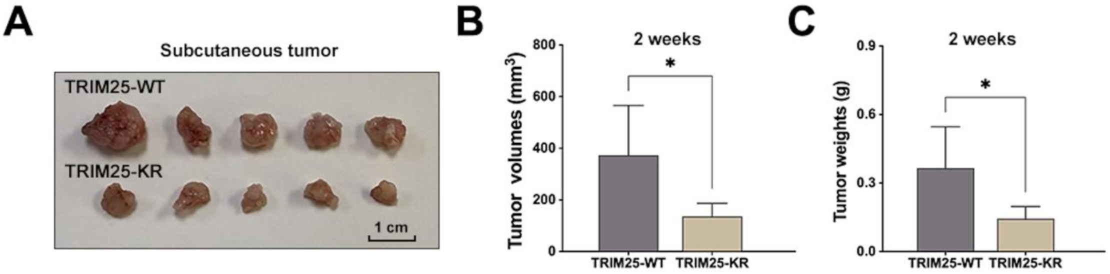

Fig. S9 Deacetylation modification of TRIM25 attenuates tumor growth *in vivo* compared to wild type. (A) A549 cells stably overexpressing TRIM25-WT or TRIM25-K392R (3×10^6 cells/mouse) were injected into the right flank of mice, and after 2 weeks, the nude mice were euthanized and the tumors were surgically removed and photographed. (B-C) Tumor volume and weight were compared between the TRIM25-WT group and the TRIM25-K392R group.

Supplementary Fig. S10

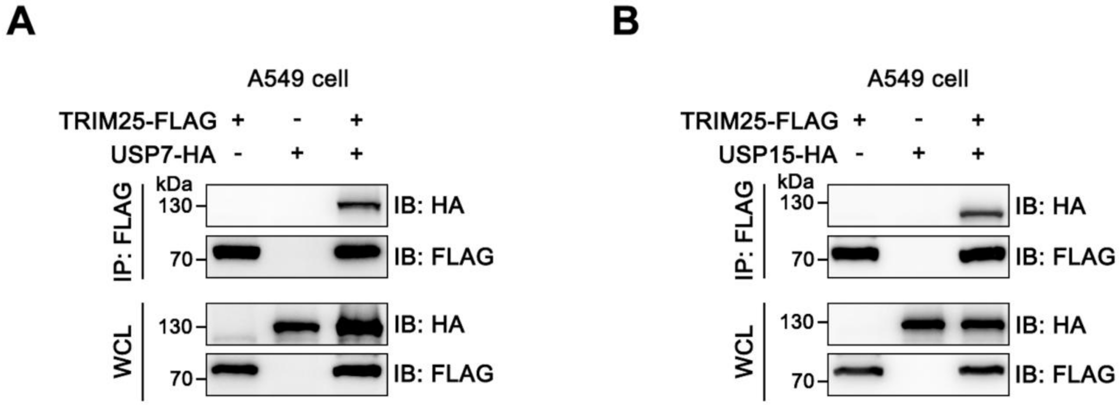

Fig. S10 TRIM25 interacts with USP7 and USP15 at the exogenous level in NSCLC cells. (A) TRIM25-FLAG and USP7-HA plasmids were transfected into A549 cells, and co-IP assays were performed with anti-FLAG magnetic beads to detect exogenous interactions. (B) TRIM25-FLAG and USP15-HA plasmids were transfected into A549 cells, and co-IP assays were performed with anti-FLAG magnetic beads to detect exogenous interactions.

Supplementary Fig. S11

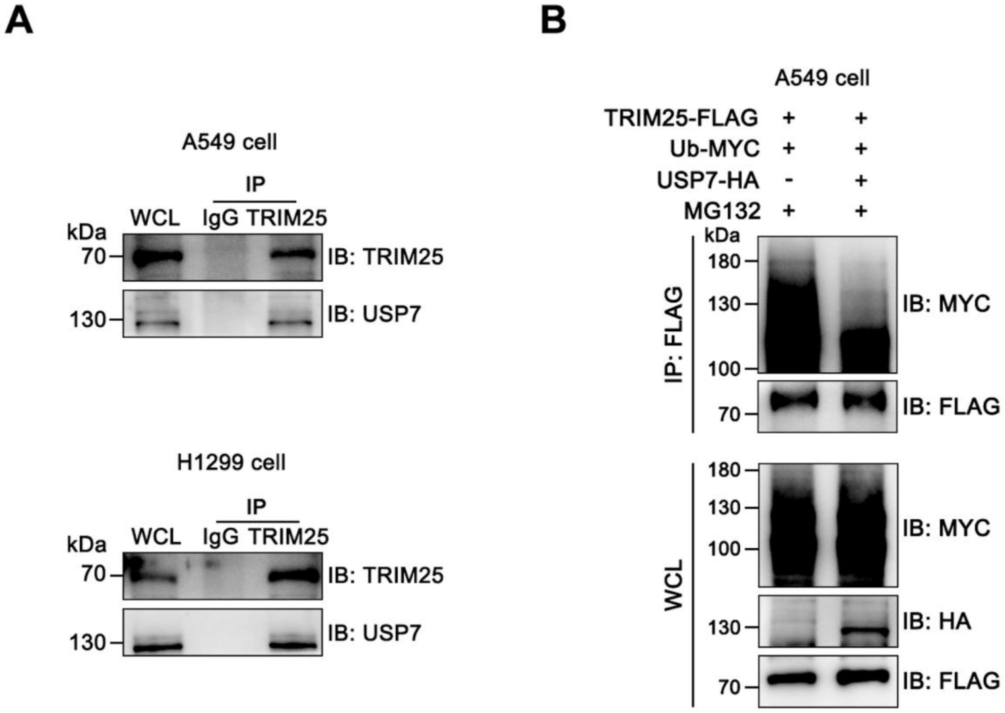

Fig. S11 USP7 endogenously interacts with TRIM25 and mediates its deubiquitination in NSCLC cells. (A) In A549 and H1299 cells, the anti-TRIM25 antibody was used for endogenous IP to detect the interaction between USP7 and TRIM25. (B) In A549 cells, plasmids were transfected as indicated and IP was performed using anti-FLAG magnetic beads, followed by western blot experiments.
