# Supplementary material for: Acetylation-dependent USP7-TRIM25 axis drives oncogenic progression in non-small cell lung cancer
Source: Cell Death Dis. 2025 Oct 6;16(1):695. doi: 10.1038/s41419-025-08034-9 (PMC12501092; doi:10.1038/s41419-025-08034-9)
Supplement: Supplementary file 2 — Supplementary Table S1 [file 41419_2025_8034_MOESM2_ESM.pdf]

Table S1. Primers used for plasmid construction, qRT-PCR, as well as sequences of shRNAs and siRNAs.

**Primers for plasmid construction**	**Sequence**
HA-TRIM25-△RING-F	acctccatagaagattctagaATGGCCGTCTACCAGGCG
HA-TRIM25-△RING-R	aacatcgtatgggtaggatccCTTGGGGGAGCAGATGGAG
HA-TRIM25-△RBB-F	acctccatagaagattctagaATGTCGAGAGCACTGGATGATGT
HA-TRIM25-△RBB-R	aacatcgtatgggtaggatccCTTGGGGGAGCAGATGGAG
HA-TRIM25-△RBBM-F	acctccatagaagattctagaATGAAGGTGCTGGAGACCTTCC
HA-TRIM25-△RBBM-R	aacatcgtatgggtaggatccCTTGGGGGAGCAGATGGAG
HA-TRIM25-△SPRY-F	acctccatagaagattctagaATGGCAGAGCTGTGCCCC
HA-TRIM25-△SPRY-R	aacatcgtatgggtaggatccGGCCTTGAGAGATGTTGAGTTCG
**Primers for qRT-PCR**	**Sequence**
β-actin-F	CACAGAGCCTCGCCTTTGCC
β-actin-R	CATGCCGGAGCCGTTGTCG
TRIM25-F	AATCGGCTGCGGAATTTTTC
TRIM25-R	TCTCACATCATCCAGTGCTCT
**shRNAs**	**Sequence**
sh-NC	TTCTCCGAACGTGTCACGTTT
sh-TRIM25-1	GGGATGAGTTCGAGTTTCTGG
sh-TRIM25-2	TTCCTCTTAGAGAAAATCCAT
**siRNAs**	**Sequence**
si-SIRT7	GAACGGAACTCGGGTTATT
si-CBP	GGCCTCCTCAATAGTAACT
si-NC	TTCTCCGAACGTGTCACGTTT
