# Supplementary material for: Acetylation-dependent USP7-TRIM25 axis drives oncogenic progression in non-small cell lung cancer
Source: Cell Death Dis. 2025 Oct 6;16(1):695. doi: 10.1038/s41419-025-08034-9 (PMC12501092; doi:10.1038/s41419-025-08034-9)

Source data for Figure 2

Fig. 2A

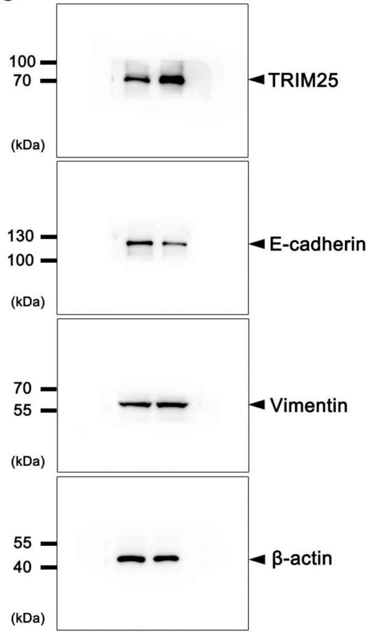

Fig. 2B

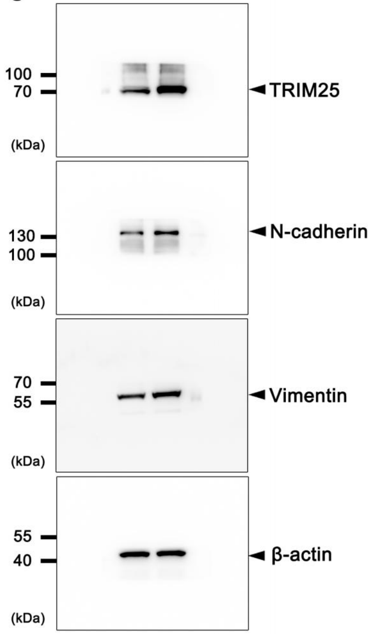

Fig. 2C

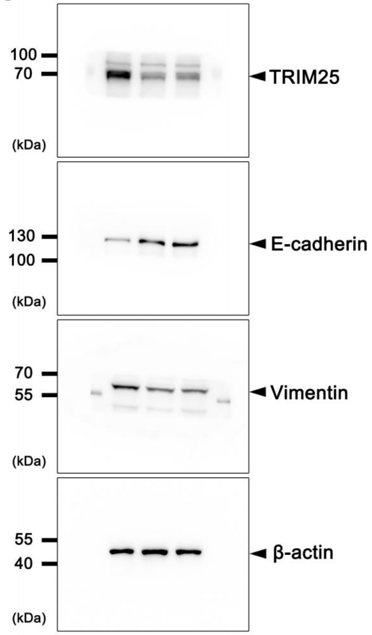

Fig. 2D

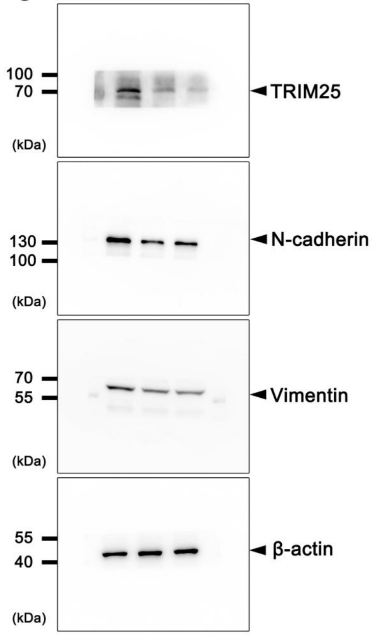

Source data for Figure 3

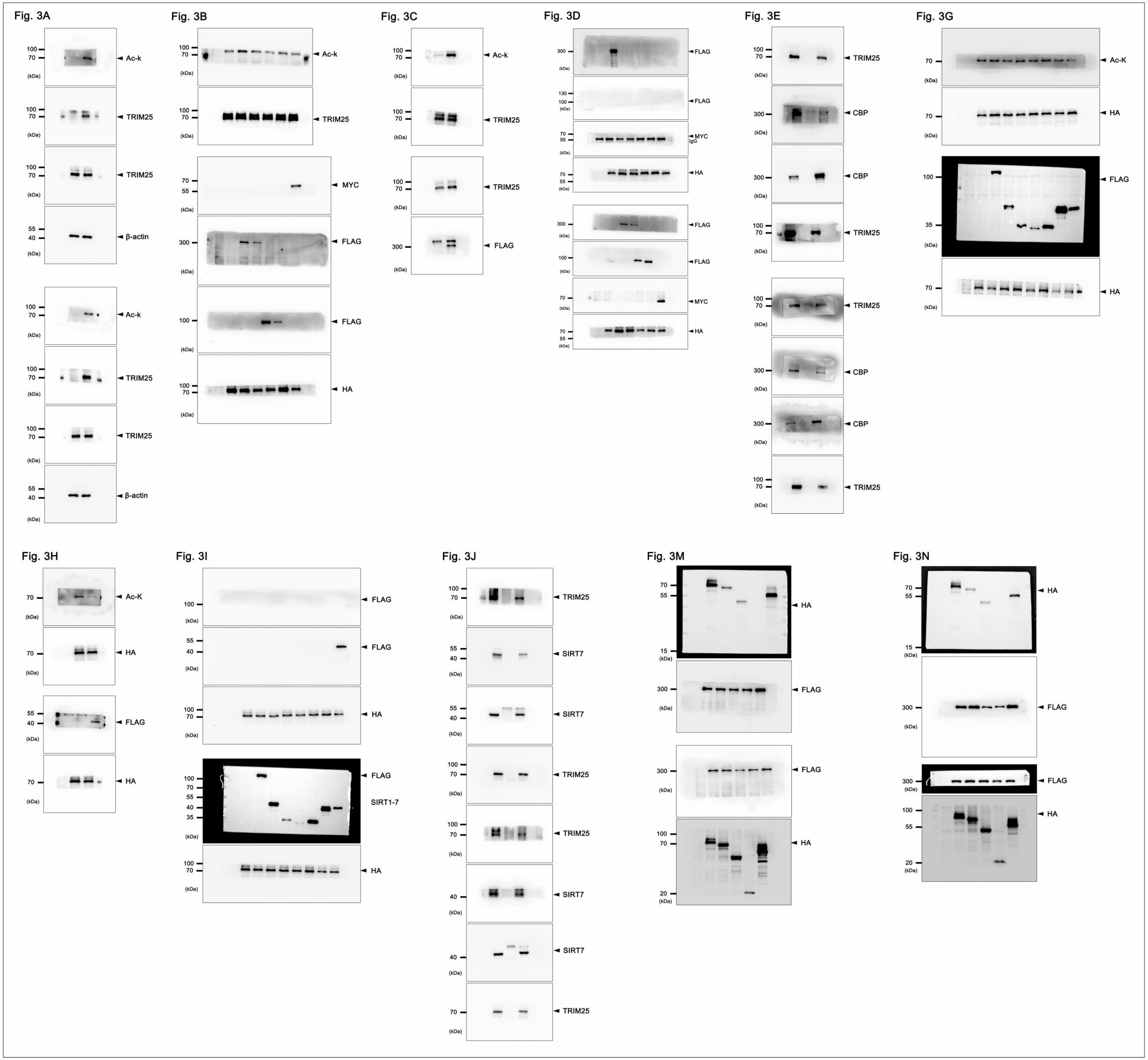

Source data for Figure 4

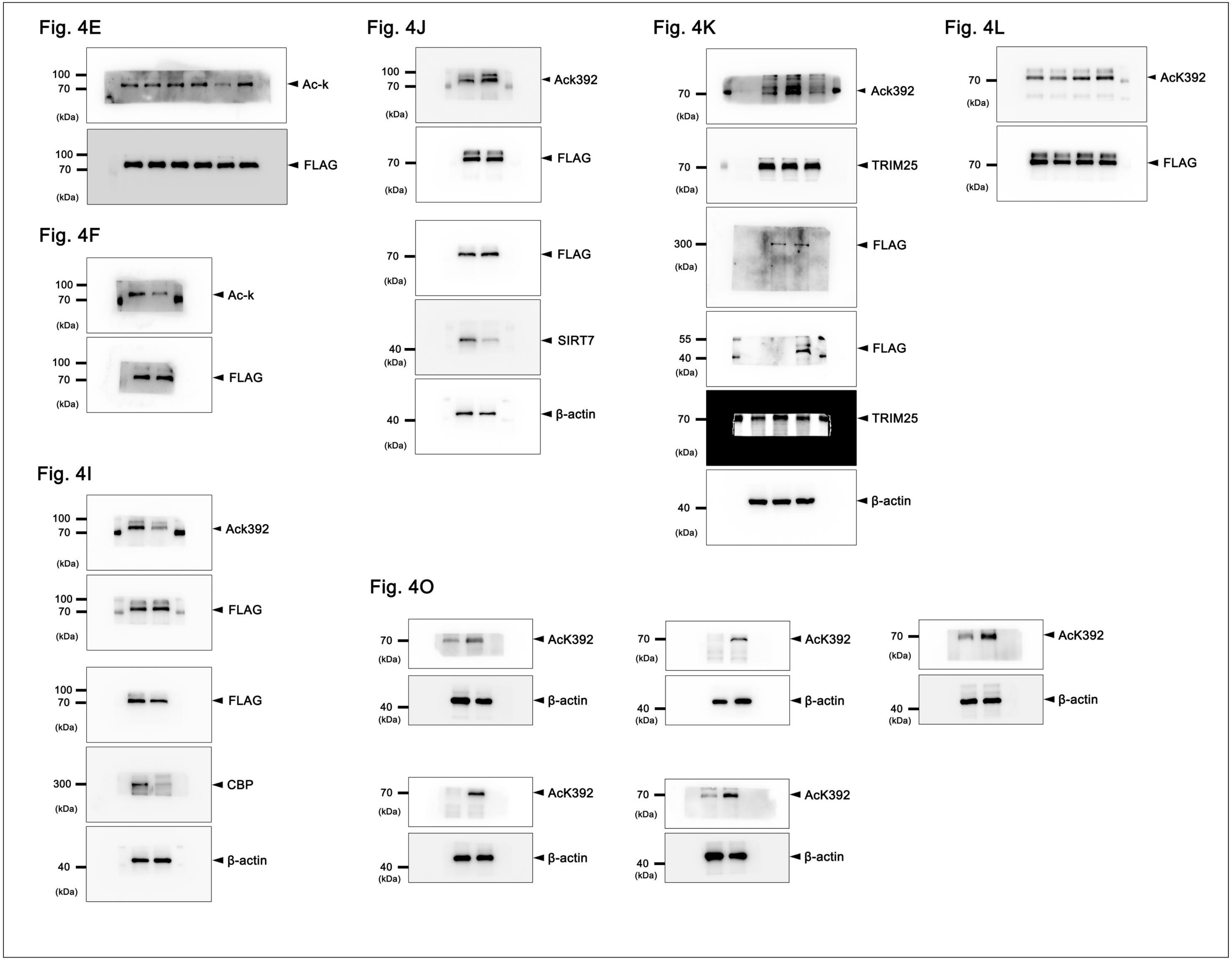

Source data for Figure 5

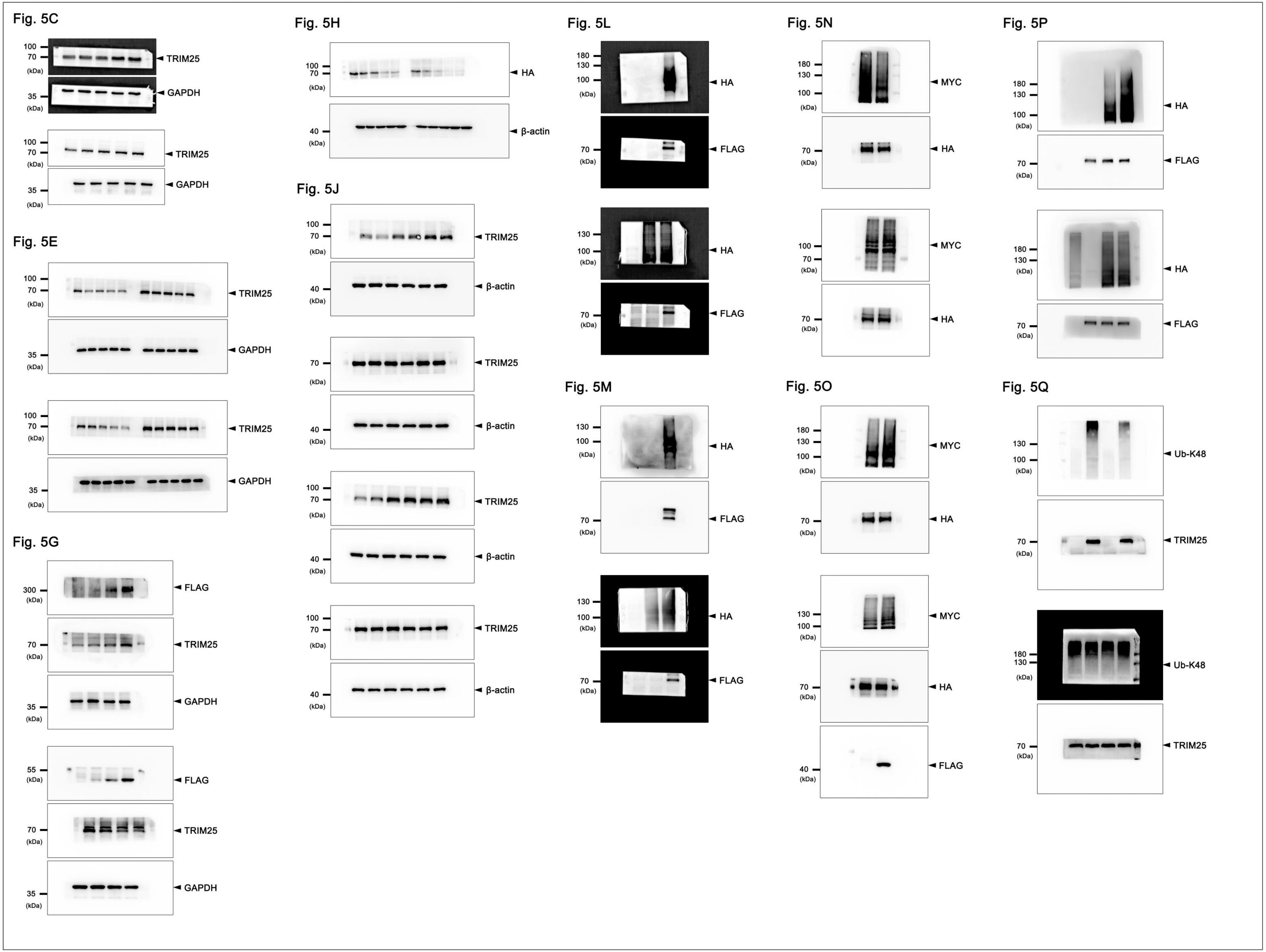

Source data for Figure 7

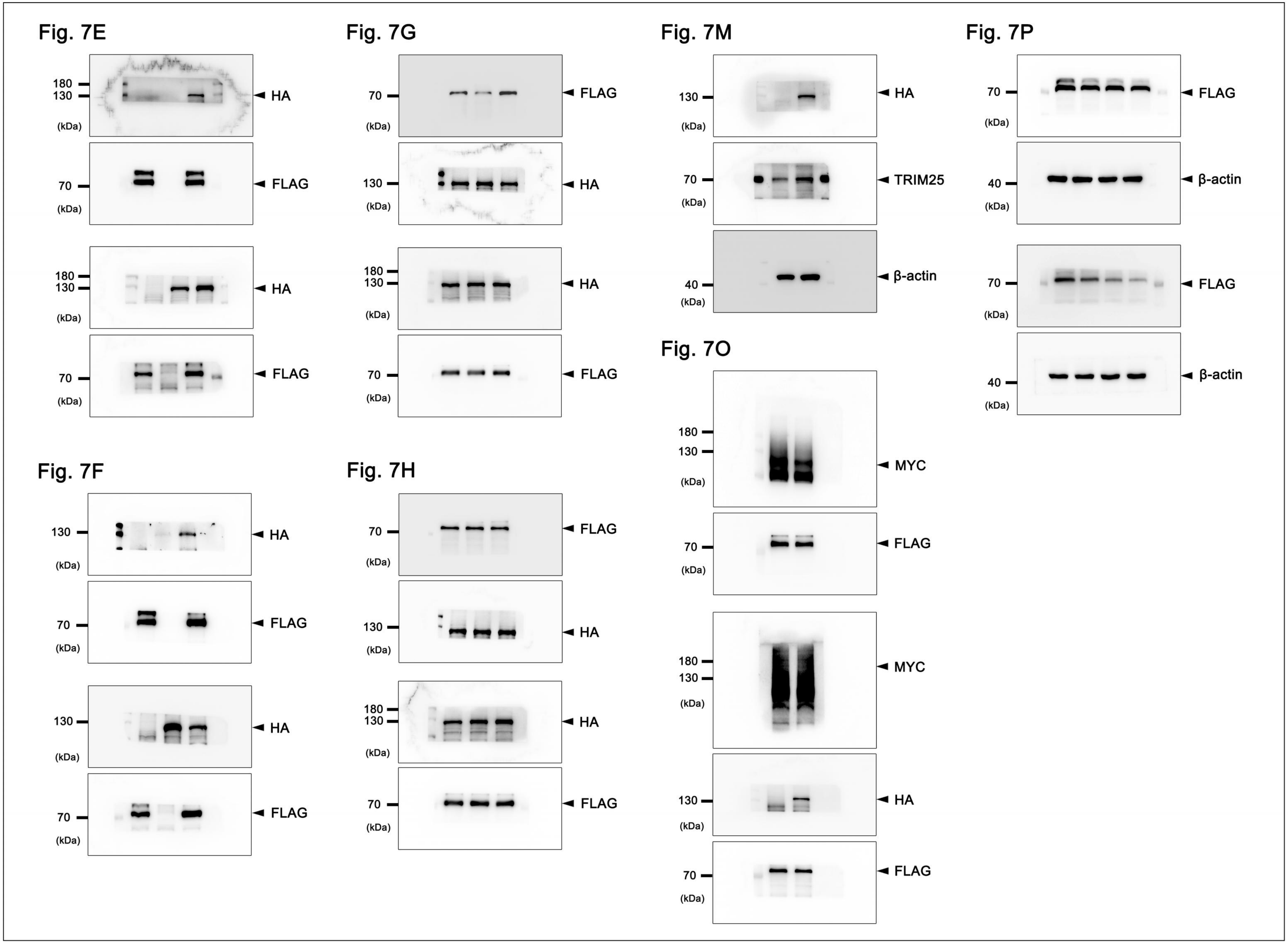

Source data for Figure S1

Fig. S1A

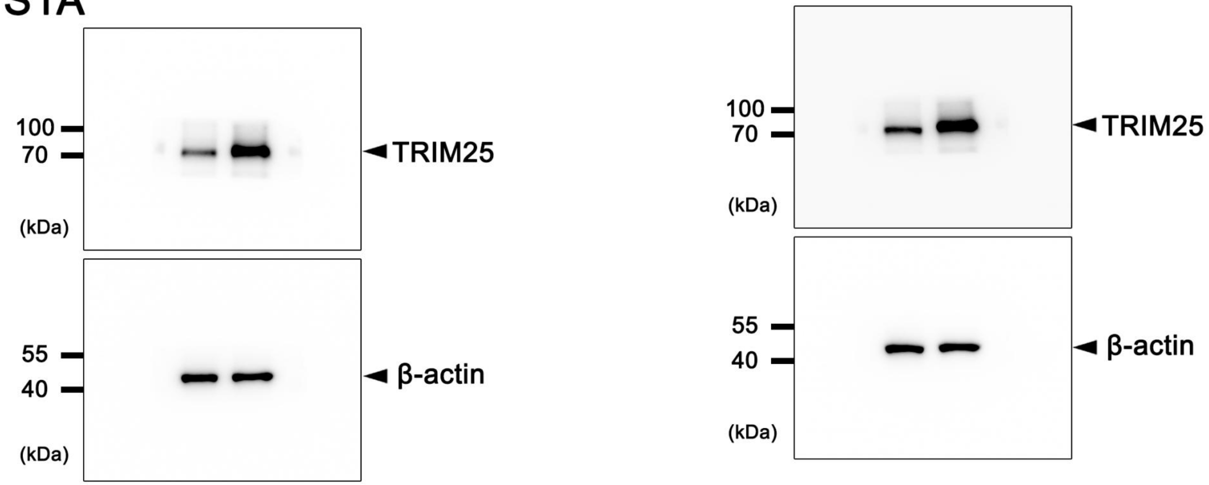

Fig. S1B

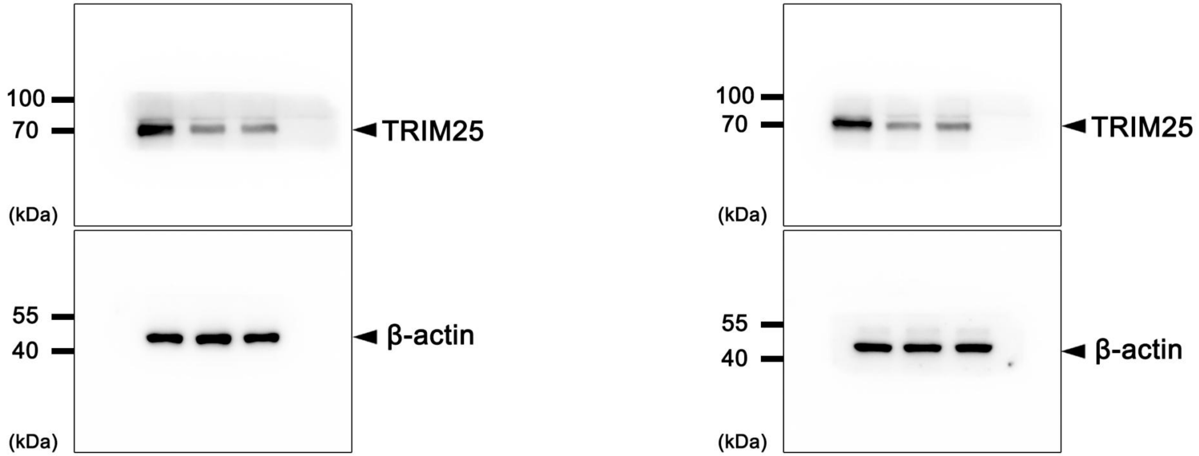

Source data for Figure S3-5

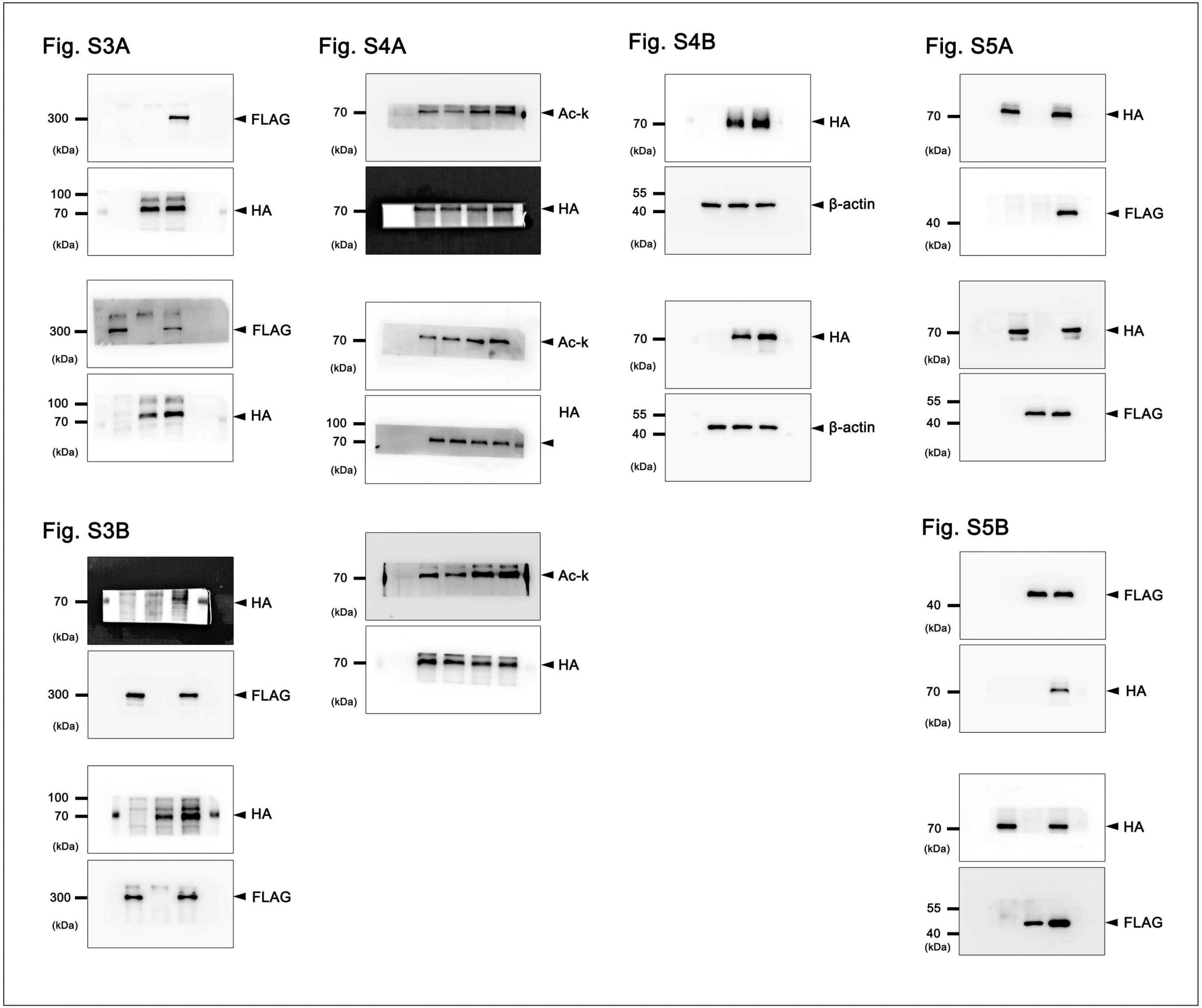

Source data for Figure S7-8

Fig. S7A

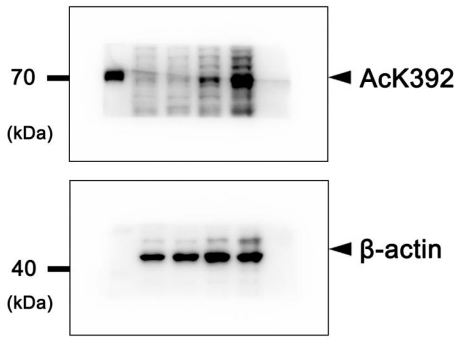

Fig. S8A

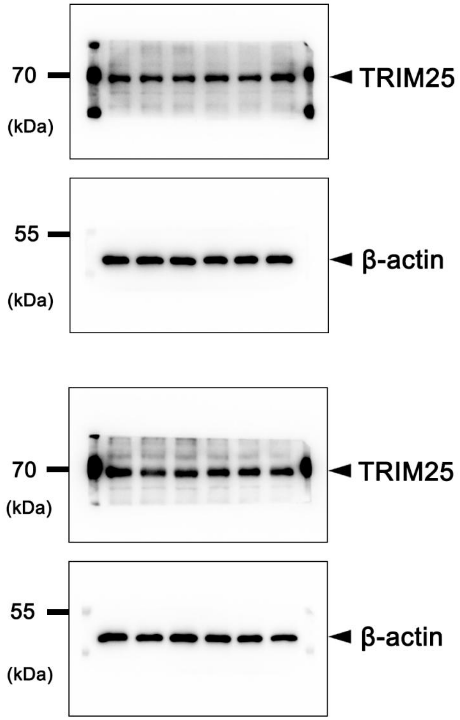

Fig. S8B

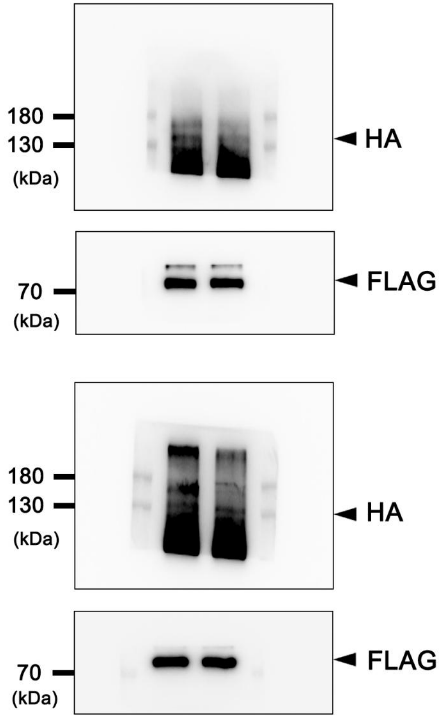

Source data for Figure S10-11

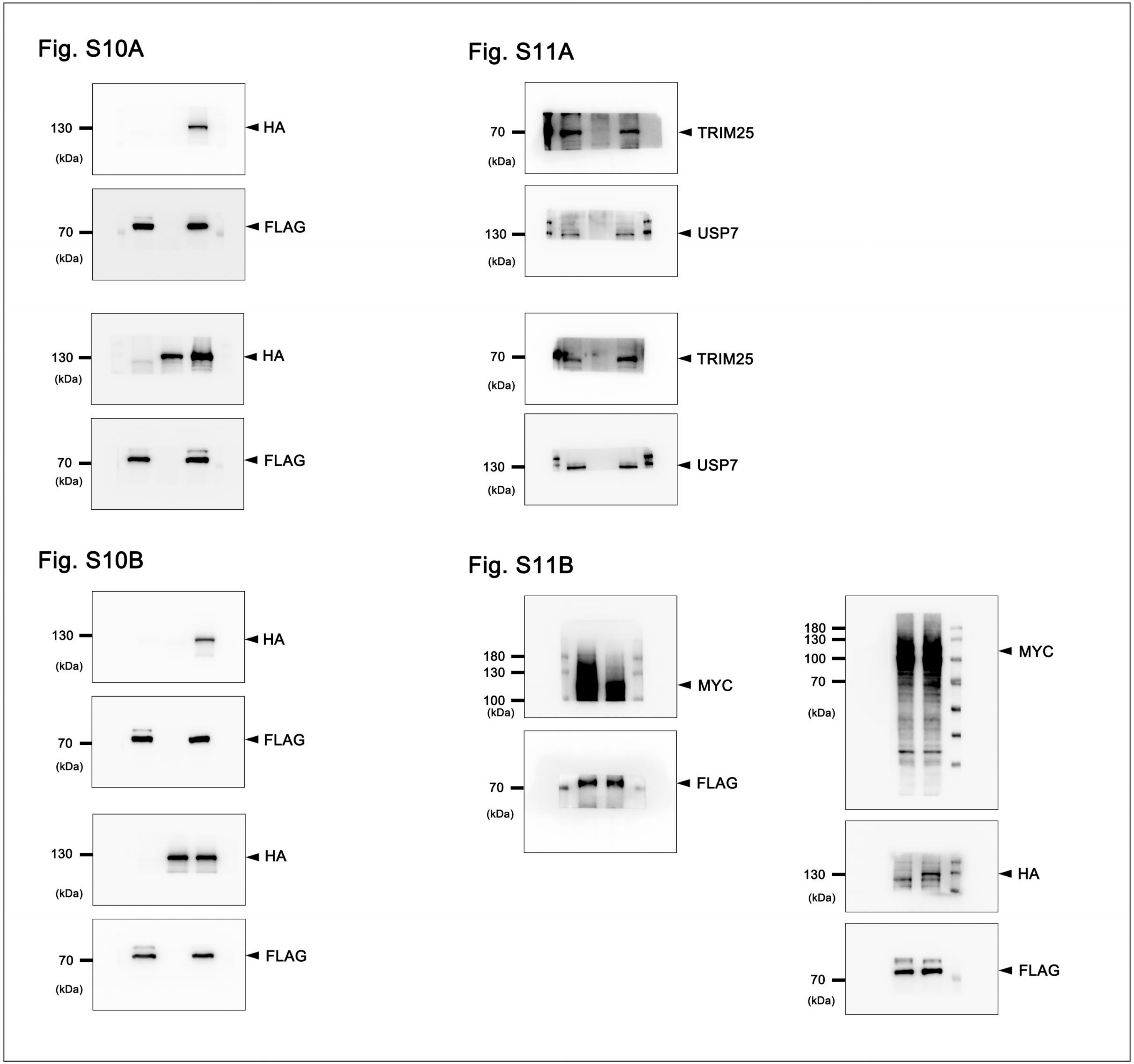

Supplement: Supplementary file 3 — Source data for Figures [file 41419_2025_8034_MOESM3_ESM.pdf]
